# Population Genomics Provides Novel Insights Into Evolutionary Relationships and Local Adaptation of Two Ecotypes *Coilia nasus*

**DOI:** 10.1002/ece3.72815

**Published:** 2025-12-23

**Authors:** Fengjiao Ma, Sheng Wang, Wenzhi Ma, Hui Wang, Haotian Jin, Legen Peng, Guojun Yin, Kai Liu

**Affiliations:** ^1^ Wuxi Fisheries College Nanjing Agricultural University Wuxi China; ^2^ Key Laboratory of Freshwater Fisheries and Germplasm Resources Utilization, Ministry of Agriculture and Rural Affairs, Freshwater Fisheries Research Center, Chinese Academy of Fishery Sciences Wuxi China; ^3^ Aquatic Conservation and Rescue Center of Jiangxi Province Nanchang China

**Keywords:** *Coilia nasus*, ecotypes, evolutionary relationships, local adaptation, whole‐genome resequencing

## Abstract

*Coilia nasus* is an economically important fish species that exhibits two distinct life history strategies: an anadromous form, which migrates from seawater to freshwater to spawn, and a freshwater‐resident form that completes its entire life cycle in freshwater habitats. The taxonomic status of the freshwater‐resident form (*C. nasus taihuensis*) has long remained unresolved, as it is unclear whether it represents a distinct subspecies or a divergent ecotype. To explore the evolutionary relationships between anadromous and freshwater‐resident forms, we performed whole‐genome resequencing of 12 geographical populations from the Yangtze River, Huaihe River, and Yellow River systems and obtained 8,701,537 high‐quality single nucleotide polymorphisms (SNPs). Based on multiple analyses (NJ tree, PCA, and ADMIXTURE analysis), our study confirmed that *C. nasus taihuensis* is not a valid subspecies but rather a freshwater ecotype of *C. nasus* . Compared to anadromous *C. nasus* , freshwater‐resident *C. nasus taihuensis* exhibited slightly higher genetic diversity, an elevated Tajima's *D* value, and lower levels of linkage disequilibrium, suggesting potential differences in demographic history and selective pressures. The estimated divergence time of approximately 4.6 thousand years ago between the two ecotypes coincides with the geological formation of Tai Lake. Whole‐genome scans identified 290 selective sweep genes that underlie local adaptation in anadromous *C. nasus* . Several candidate genes were mainly involved in energy metabolism (*SCD*, *HOAD*, and *DEGS2*) and osmoregulation (*CLCN4*, *IGF2*, and *LRP1*), suggesting their potential contribution to highly efficient long‐distance migration and adaptation to seawater. In contrast, freshwater‐resident *C. nasus taihuensis* exhibited strong signatures of selection in genes related to osmotic and ionic balance (*ATP1A1*, *AQP9*, *NBC*, *NHE3*, and *CA4*), which may have driven the genetic divergence of this ecotype. Our comprehensive study provides valuable insights into the genetic basis of *C. nasus* , with critical implications for further conservation efforts of this ecologically valuable fish.

## Graphical abstract

Based on whole‐genome resequencing, we determined that the freshwater‐resident form ( *C. nasus* taihuensis) is an ecotype of anadromous *Coilia nasus* , not a distinct subspecies. The two ecotypes diverged approximately 4.6 thousand years ago, coinciding with the formation of Tai Lake. Genome scans revealed distinct selective sweeps, with anadromous fish showing adaptations for energy metabolism and osmoregulation for seawater, while the freshwater ecotype shows selection in genes for maintaining osmotic balance in freshwater.

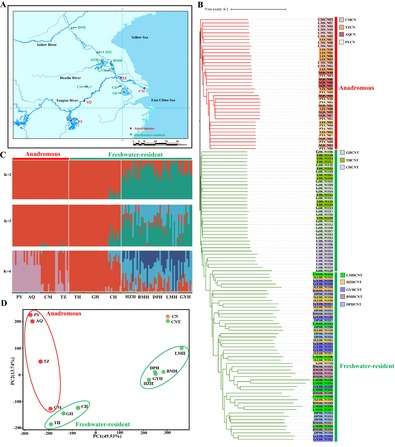

## 1 Introduction

The Japanese grenadier anchovy ( *Coilia nasus* ) is an ecologically important anadromous fish in China. It mainly inhabits the coastal waters of the Northwest Pacific and migrates into rivers connected to the sea, including the Yangtze River, the Yellow River, and Qiantang River (Xuan et al. 2021). Every spring, anadromous *C. nasus* undertakes long‐distance migrations between its spawning and feeding grounds, primarily along the Yangtze River, to spawn either in the main stream or in river‐connected lakes such as Poyang Lake (Ying et al. 2020). *C. nasus* exhibits two distinct life history strategies: anadromy and freshwater residency (Cheng et al. 2019). Anadromous individuals migrate from freshwater to sea before returning to natal areas for spawning. In contrast, non‐anadromous individuals (known as *Coilia nasus taihuensis*) remain exclusively in freshwater environments throughout their lives (Gao et al. 2021). These divergent strategies reflect significant local adaptation, which has been documented in recent studies (Zong et al. 2021). Environmental conditions can differ among populations and impose differential selective pressures on life‐history traits. The ability to migrate between saltwater and freshwater environments involves morphological, behavioral, and physiological adaptations (Folmar and Dickhoff 1980; Hubert et al. 2007; Seear et al. 2010; Boulet et al. 2012). Several of these traits have been shown to be highly heritable, which supports potential for evolutionary divergence among populations (Duston et al. 2011). Recent advances in high‐resolution, genome‐wide studies now allow us to improve our understanding of the genetic mechanisms underlying differentiation between resident and anadromous populations of *C. nasus* .

Following deglaciation after the last glacial maximum (< 20,000 years ago), new freshwater lakes formed, such as Taihu Lake, Gehu Lake, Hongze Lake, and Dongping Lake. While most populations of *C. nasus* are anadromous, some have become landlocked in freshwater habitats due to factors such as isostatic rebound and physical barriers. Once isolated, these populations adapted to a fully freshwater life cycle, completing all developmental stages without migrating to the sea (Delgado et al. 2019). The loss of anadromy leads to predictable evolutionary consequences (Delgado et al. 2020). For example, anadromous *C. nasus* and freshwater‐resident *C. nasus taihuensis* consistently differ in morphology, feeding ecology, reproductive behavior, and salinity tolerance (Gao et al. 2021). Freshwater‐resident individuals experience different selective pressures compared to their anadromous counterparts, resulting in physiological adaptations in osmoregulation and swimming performance (Velotta et al. 2014, 2018). Similarly, landlocked salmon populations exhibit reduced selection pressure on traits adapted to seawater (Kjærner‐Semb et al. 2021). Previous studies have confirmed that key traits in *C. nasus* have high heritability, allowing for divergent selection between resident and migratory ecotypes under differing environmental conditions (Zong et al. 2021). However, the genomic mechanisms underlying adaptive divergence between anadromous and resident populations in *C. nasus* remain poorly understood.

The genetic structure of *C. nasus* populations has attracted considerable scientific and public attention owing to its high socio‐economic value and the urgent need for effective management strategies. However, early studies were constrained in scope, focusing on only a few populations or genetic markers (Xue et al. 2019). Numerous molecular investigations, primarily based on mtDNA, subsets of SNPs, and microsatellite loci, have offered valuable insights into the population structure and genetic diversity of *C. nasus* (Cheng et al. 2011; Gao et al. 2020; Li et al. 2023; Zhang et al. 2023). Despite ongoing research, the taxonomic status of freshwater resident *C. nasus taihuensis* remains unresolved, and the genetic relationships between anadromous *C. nasus* and freshwater resident *C. nasus taihuensis* remain poorly understood (Xue et al. 2019). To date, no comprehensive study has elucidated the genetic structure and relationships among *C. nasus* populations across large geographic scales using whole‐genome SNP markers. Recent advances in population genomics have provided powerful tools to explore the genetic basis of differentiation between anadromous *C. nasus* and freshwater resident *C. nasus taihuensis*. Therefore, clarifying the population genetic structure and evolutionary relationships between two ecotypes through whole‐genome resequencing and range‐wide sampling is of critical importance. Genome‐wide selective scans now make it possible to identify genetic variation associated with local adaptation (Vitti et al. 2013). To explore the evolutionary relationships and genetic adaptations of two ecotypes with distinct life histories, we performed whole‐genome resequencing of 128 *C. nasus* individuals. The study aimed to: (i) characterize genomic diversity and population differentiation between freshwater and anadromous populations; (ii) elucidate the evolutionary history and gene flow of the *C. nasus* species; and (iii) detect signatures of selection and identify candidate genes and associated biological functions underlying adaptive processes. Our study provides foundational genomic resources for *C. nasus* and offers critical insights for the management and conservation of its wild populations.

## 2 Materials and Methods

### 2.1 Sample Collection and Microchemical Analysis

**FIGURE 1:**
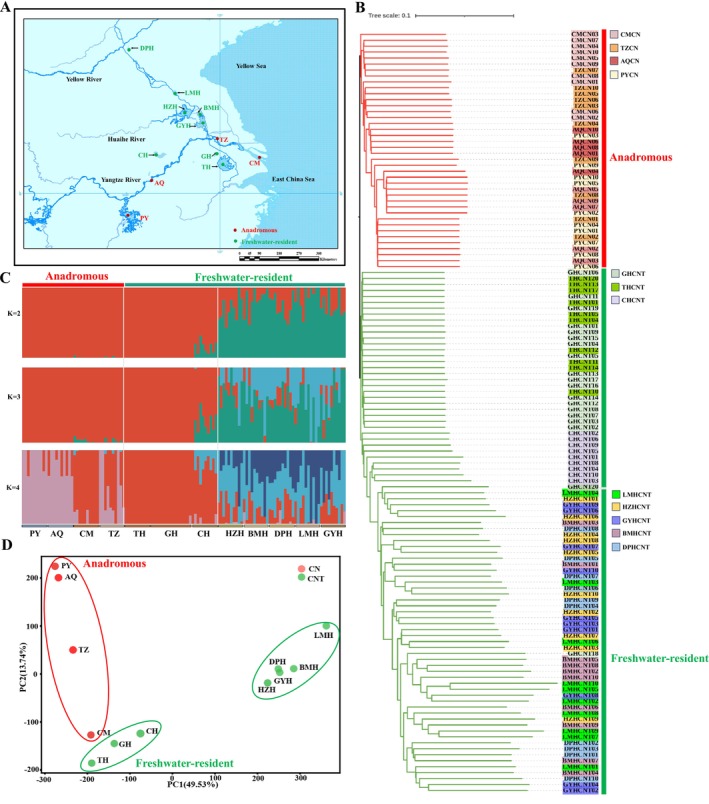
Population genetic structure of anadromous *C. nasus* and freshwater‐resident *C. nasus taihuensis*, based on whole‐genome SNPs. (A) Geographic distribution of sampling locations, with circles colored according to ecotypes. (B) Neighbor‐joining (NJ) phylogenetic tree constructed using whole‐genome SNPs data. Each color corresponds to a distinct population, and the scale bar represents the genetic distance between individuals. (C) Population structure analysis for *K* values ranging from 2 to 4. Each bar represents an individual, with segment colors indicating the proportional ancestry from each inferred ancestral population. (D) Principal component analysis (PCA) of 12 geographical distinct populations. Abbreviations: CM, Chongming section; TZ, Taizhou section; AQ, Anqing section; PY, Poyang Lake; GH, Gehu Lake; TH, Taihu Lake; CH, Chaohu Lake; HZH, Hongze Lake; LMH, Luoma Lake; BMH, Baima Lake; GYH, Gaoyou Lake; DPH, Dongping Lake.

For comparative population genomics analysis, a total of 128 *C. nasus* samples were collected using gill nets between 2022 and 2023, representing 12 distinct geographical populations. Among these, 40 individuals of anadromous *C. nasus* were collected from four sampling locations in the lower reaches of the Yangtze River and its conjoining freshwater lakes: Chongming section (CM), Taizhou section (TZ), Anqing section (AQ), and Poyang Lake (PY). Additionally, 88 individuals of freshwater‐resident *C. nasus taihuensis* were collected from eight freshwater lakes, including Chaohu Lake (CH), Taihu Lake (TH), Gehu Lake (GH), Hongze Lake (HZH), Baima Lake (BMH), Gaoyou Lake (GYH), Luoma Lake (LMH), and Dongping Lake (DPH). The sample information is summarized in Table 1 and Figure 1A. As indicated in a previous study, otolith microchemistry analysis serves as an effective method for elucidating the life history traits of *C. nasus* (Jiang et al. 2022). In this study, the left sagittal otolith of *C. nasus* was selected for microchemical analysis, with Sr/Ca ratios expressed as Sr/Ca × 1000. All specimens were classified based on morphological characteristics (supermaxilla/head length > 1) and the Sr/Ca ratios of their sagittal otoliths: individuals with a Sr/Ca × 1000 ratio greater than 3 were identified as *C. nasus* , whereas those with a ratio less than 3 were identified as *C. nasus taihuensis*. The sampling process was conducted in strict accordance with applicable Chinese laws and ethical guidelines governing experimental procedures. Muscle tissues were collected from each sample, immediately stored in liquid nitrogen, and subsequently maintained at −80°C until further analysis.

**TABLE 1:** Sampling information and identification results of ecotypes among 12 distinct geographical populations of *Coilia nasus* in the present study.

Population	River basin	Sample location	Sample ID	Date of collection	Sample size (*n*)	Life history forms estimated by otolith microchemistry of Sr and Ca
*Coilia nasus*	Yangtze River	Chongming section	CM	June 24, 2022	10	Anadromous
	Yangtze River	Taizhou section	TZ	May 31, 2022	10	Anadromous
	Yangtze River	Anqing section	AQ	May 22–23, 2022	10	Anadromous
	Poyang Lake	Poyang Lake	PY	September 24, 2022	10	Anadromous
*Coilia nasus taihuensis*	Yangtze River	Chaohu Lake	CH	July 25, 2022	9	Freshwater‐resident
	Yangtze River	Taihu Lake	TH	April 12, 2023	10	Freshwater‐resident
	Yangtze River	Gehu Lake	GH	October 15, 2022	19	Freshwater‐resident
	Huaihe River	Hongze Lake	HZH	September 29, 2022	10	Freshwater‐resident
	Huaihe River	Baima Lake	BMH	September 25, 2022	10	Freshwater‐resident
	Huaihe River	Gaoyou Lake	GYH	September 22, 2023	10	Freshwater‐resident
	Huaihe River	Luoma Lake	LMH	August 31, 2023	10	Freshwater‐resident
	Yellow River	Dongping Lake	DPH	August 1, 2023	10	Freshwater‐resident
Total					128	

### 2.2 Genome Resequencing and SNP Calling

High‐quality genomic DNA was extracted from the muscle tissues of all samples using the DNeasy Blood & Tissue Kit (Qiagen, Valencia, CA, USA) according to the manufacturer's protocol. The integrity of the extracted DNA was evaluated using 1% agarose gel electrophoresis. DNA concentrations from 128 individuals were quantified by Qubit Fluorometer (Invitrogen, USA). For each sample, DNA libraries with an insert size of 350 bp were constructed and sequenced on the DNBSEQ‐T7 platform provided by BGI Genomics (Wuhan, China), producing 150‐bp paired‐end reads. The raw sequencing data were subjected to quality filtering in order to retain only high‐quality reads for subsequent analysis. In a prior study, a gap‐free chromosome‐level genome assembly of anadromous *C. nasus* was completed, with a total genome size of 851.67 Mb and a contig N50 of 35.42 Mb, providing a high‐quality reference genome for this study (Ma et al. 2023). Clean reads were mapped to the *C. nasus* reference genome (GCA_027475355.1, NCBI) using the Burrows‐Wheeler Aligner (BWA) software (v 0.7.17) with the MEM algorithm (Li and Durbin 2009). The resulting alignment files were sorted and converted into BAM format using SAMtools (v1.9). The Picard software (v 2.5.0) (https://broadinstitute.github.io/picard) was used to remove putative PCR duplicates and to calculate genomic coverage and sequencing depth. Subsequently, the HaplotypeCaller module of GATK (v 4.1) was applied to generate a genomic variant call format file (gVCF) for each sample (McKenna et al. 2010). Raw single nucleotide polymorphisms (SNPs) were filtered using VarianFilteration tool in GATK with the following parameters: —filter‐expression ‘QD < 2.0||MQ < 40.0||FS > 60.0||SOR > 3.0||MQRankSum < −12.5||ReadPosRankSum < −8.0’—filter‐name ‘SNP_filter’. Following this, low‐quality SNPs were further filtered using VCFtools (v 0.1.16) with parameters: ‐‐max‐missing 0.9 ‐‐maf 0.05 ‐‐min‐meanDP 5 ‐‐min‐alleles 2 ‐‐max‐alleles 2 (Danecek et al. 2011). Finally, only SNPs satisfying the stringent criteria of minor allele frequency (MAF) > 0.05, coverage depth > 5× and presence in at least 90% of the samples were retained for the subsequent population genetic analysis.

### 2.3 Phylogenetic Tree and Population Structure

For phylogenetic analysis, a genetic distance matrix was constructed for 128 individuals using VCF2Dis v1.47 (https://github.com/BGI‐shenzhen/VCF2Dis). Based on whole‐genome SNP data, an individual‐based neighbor‐joining (NJ) tree was constructed using the computed distance matrix and visualized using MEGA (v7.0.14; https://www.megasoftware.net/) and the online tool iTOL (https://itol.embl.de/). SNPs for linkage disequilibrium (LD) were filtered using PLINK v1.9 with the command “‐‐indep‐pairwise 50 10 0.2”. Principal component analysis (PCA) was conducted to assess population genetic structure using PLINK (v1.9) and GCTA (v1.92.2) software (Purcell et al. 2007), with the first two principal components labeled as PC1 and PC2, respectively. Population structure and ancestry proportions were analyzed using the ADMIXTURE program (v1.3.0) based on the filtered SNPs dataset. The optimal number of genetic clusters (*K*) was determined by calculating cross‐validation error (CV). *K* values ranging from 2 to 5 were tested, and the *K* with the lowest CV error was selected. Population structure and PCA plots were drawn using R (v3.6.3, https://www.r‐project.org/).

### 2.4 Genetic Diversity and Differentiation

The nucleotide diversity (*π*) was calculated using VCFtools (v0.1.16) with a 10 kb sliding window. A neutrality test based on Tajima's *D* was also conducted using the same sliding windows (Danecek et al. 2011). To evaluate genome‐wide differentiation between the two ecotypes, the genetic differentiation index (*F*st) was computed using VCFtools (v 0.1.16) with 10 kb non‐overlapping sliding windows (Charlesworth 1998; Ru et al. 2018). Genetic differentiation levels were categorized as follows: *F*st < 0.05 indicated negligible differentiation, 0.05 < *F*st < 0.15 indicated moderate differentiation, 0.15 < *F*st < 0.25 indicated substantial differentiation, *F*st > 0.25 indicated extreme differentiation. Linkage disequilibrium (LD) decay was assessed by calculating the correlation coefficients (*r* ^2^) for all pairs of SNPs using PopLDdecay v 3.42 (https://github.com/BGI‐shenzhen/PopLDdecay) (Zhang et al. 2019).

### 2.5 Demographic History and Gene Flow

To reconstruct the demographic history of anadromous *C. nasus* and freshwater‐resident *C. nasus taihuensis*, we applied the Sequentially Markovian Coalescent (SMC++) method to estimate effective population size (*Ne*) and divergence time (Terhorst et al. 2017). For this purpose, all individuals were selected from each geographical population. First, we used VCF2smc to convert VCF files into the required SMC++ input format. Then, we employed the estimate command to infer a detailed population size history. Furthermore, we utilized the split command to model clear divergence events between the two populations, using marginal estimates obtained from the previous step. Given that the mutation rate for *Coilia* species is currently unknown, we adopted a neutral mutation rate (*μ*) of 2.91 × 10^−9^ per site per generation (Pettersson et al. 2019). The generation time was calculated as twice the age at sexual maturity, and the generation time (*g*) was set to 3.1 years, obtained from FishBase Database (https://fishbase.net.br/summary/). In addition, TreeMix was employed to detect potential migration events among 12 geographical populations from different sampling locations. A series of simulations were conducted to determine the optimal number of migration edges in the TreeMix analysis, with the number of migration edges ranging from 0 to 10, and each scenario was replicated 10 times. The results were subsequently analyzed using the online version of OptM (Fitak 2021).

### 2.6 Detection of Selective Sweeps

To identify the selective sweep regions in *C. nasus* , selective sweep analyses were performed based on three genome‐wide metrics, including nucleotide diversity ratio (*π*_CN_/*π*_CNT_, where *π*_CN_ and *π*_CNT_ were the *π* values of two populations, respectively), genetic differentiation index (*F*st) and the cross‐population composite likelihood ratio (XP‐CLR). The statistics of *F*st and *π* ratio were calculated with a sliding window size of 10 kb and a step size of 5 kb across the genome using VCFtools. Windows with *F*st values ≥ 0.18 and 5% *π* ratio (log_2_(*π*_CN_/*π*_CNT_)) were identified as candidate regions under strong selective sweeps. Specifically, regions with *F*st values in the top 5% (*F*st ≥ 0.18) and *π* ratios in the bottom 5% (log_2_(*π*_CNT_/*π*_CN_ ≤ −1.34)) were considered candidate genes for anadromous *C. nasus* . In contrast, regions with both *F*st values and *π* ratios in the top 5% (log_2_(*π*_CN_/*π*_CNT_) ≥ 0.17) were regarded as candidate genes for freshwater‐resident *C. nasus taihuensis*. The XP‐CLR method is capable of modeling allele frequency differences between two populations to detect candidate alleles that have undergone positive selection (Chen et al. 2010). For XP‐CLR analysis, the mean likelihood scores were calculated using the XP‐CLR package (https://github.com/hardingnj/xpclr) with the same sliding windows and step sizes as *F*st and *π* ratio. Genomic windows with the top 5% value of XP‐CLR were defined as divergent selective regions. The overlapping regions identified by all the three metrics were defined as the final candidate regions. Subsequently, functional analysis for candidate genes was performed against the Gene Ontology (GO) and Kyoto Encyclopedia of Genes and Genomes (KEGG) databases. We centered on those genes with significant GO terms and KEGG pathways (*p*‐values < 0.05) in corresponding lineages.

## 3 Results

### 3.1 Whole‐Genome Resequencing and Variation Calling

To enhance our understanding of the genetic relationships between anadromous *C. nasus* and freshwater‐resident *C. nasus taihuensis*, we performed whole‐genome resequencing on 128 individuals, representing 12 geographical populations across the Yangtze River, Huaihe River, and Yellow River systems. Deep resequencing of these samples yielded a total of 3.04 Tb of high‐quality clean data, with an average of 24.4 Gb per individual. The alignment achieved an average genome coverage of 90.02%, with a mean sequencing depth of 24.37× per sample (Table S1). All sequences were successfully aligned to the reference genome of anadromous *C. nasus* , with an average mapping rate of 98.36% (Table S1). Following alignment, variant detection identified 33,427,269 raw SNPs (Figure S1). After applying stringent quality filtering criteria, a total of 8,701,537 high‐quality SNPs were retained for subsequent population genetic analyses.

### 3.2 Population Structure Analyses

Using these high‐quality SNPs, we constructed a neighbor‐joining (NJ) phylogenetic tree to examine the phylogenetic relationships among the 128 samples. The NJ tree indicated that all individuals were clustered into two large clades. The PY, AQ, TZ and CM geographical populations were clustered into one clade, and the TH, GH, CH, HZH, BMH, GYH, LMH and DPH geographical populations were clustered into the other clade (Figure 1B). To further investigate the genetic structure of 12 populations from different geographic regions, we performed an ADMIXTURE analysis with ancestral group values (*K*) ranging from 2 to 4. At *K* = 2, two distinct genetic clusters were identified: one cluster included five freshwater‐resident geographical populations from the Huaihe River and Yellow River Basins (HZH, BMH, GYH, LMH, and DPH), while the other cluster consisted of seven geographical populations, including three freshwater‐resident populations (TH, GH and CH) and four anadromous populations (PY, AQ, TZ and CM) (Figure 1C). As *K* increased to 3 and 4, the TH, GH and CH populations remained as a distinct cluster, whereas the HZH, BMH, GYH, LMH, and DPH populations continued to display mixed ancestry across all *K* values. These results were supported by principal component analysis (PCA), which showed patterns consistent with those derived from the ADMIXTURE analysis. The first principal component (PC1, variance explained = 49.53%) clearly differentiated the five freshwater‐resident populations from the Huaihe River and Yellow River Basins from the remaining seven populations originating from the Yangtze River Basin (Figure 1D). Notably, the three freshwater‐resident populations (TH, GH and CH) and four anadromous populations (PY, AQ, TZ and CM) exhibit close genetic relationships. Neither PC1 nor PC2 effectively distinguished the HZH, BMH, GYH, LMH, and DPH populations, which is consistent with their mixed clustering patterns observed in the NJ tree.

### 3.3 Genetic Diversity, Differentiation, and LD Decay

**FIGURE 2:**
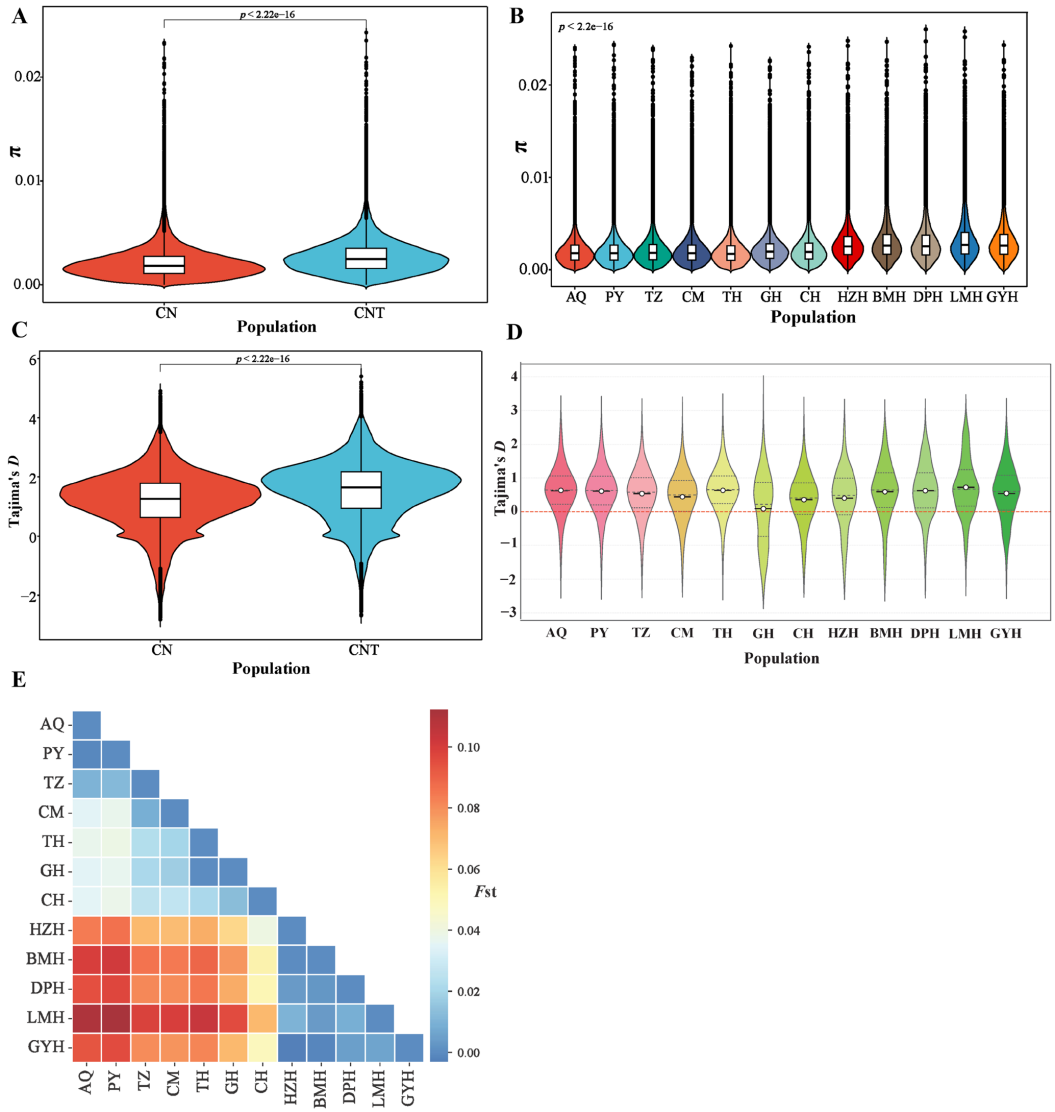
The genetic diversity and divergence among four anadromous and eight freshwater‐resident geographical populations of *Coilia nasus* . (A) Nucleotide diversity (*π*) between anadromous *C. nasus* and freshwater‐resident *C. nasus taihuensis*. (B) Nucleotide diversity (*π*) across 12 geographical populations. (C) Tajima'*D* values between anadromous *C. nasus* and freshwater‐resident *C. nasus taihuensis*. (D) Tajima'*D* values across 12 geographical populations. (E) Genetic differentiation index (*F*st) among the 12 geographical populations.

We calculated *π* and *F*st to evaluate genomic diversity and genetic divergence between anadromous *C. nasus* and freshwater‐resident *C. nasus taihuensis*. The freshwater‐resident *C. nasus taihuensis* showed slightly higher genetic diversity compared to anadromous *C. nasus* (*π*: 0.0027 ± 0.0016 vs. 0.0021 ± 0.0015, Tajima's *D*: 1.5344 vs. 1.1827) (Wilcoxon test, *p* < 2.20e‐16, Figure 2A,C). Among the eight freshwater‐resident geographical populations, TH, GH and CH exhibited lower genetic diversity (*π*: 0.0020–0.0022) compared to the remaining five populations (*π*: 0.0028–0.0031) (Figure 2B). Notably, the LMH population exhibited the highest nucleotide diversity compared to the HZH, BMH, GYH, and DPH populations (Wilcoxon test, all *p* < 2.20e‐16). We also estimated the Tajima's *D* values of different subgroups to examine possible selection patterns. Freshwater‐resident *C. nasus taihuensis* exhibited significantly higher Tajima's *D* values (1.5344 ± 0.9878) than anadromous *C. nasus* (1.1827 ± 0.9726) (Wilcoxon test, *p* < 2.22e‐16), suggesting stronger selective pressures (e.g., balancing or positive selection) acting on the former population (Figure 2C). Furthermore, the LMH population displayed significantly higher Tajima's *D* values (0.7231 ± 0.8671) than all other geographical populations, indicating distinct evolutionary pressures in Luoma Lake (Figure 2D).

**FIGURE 3:**
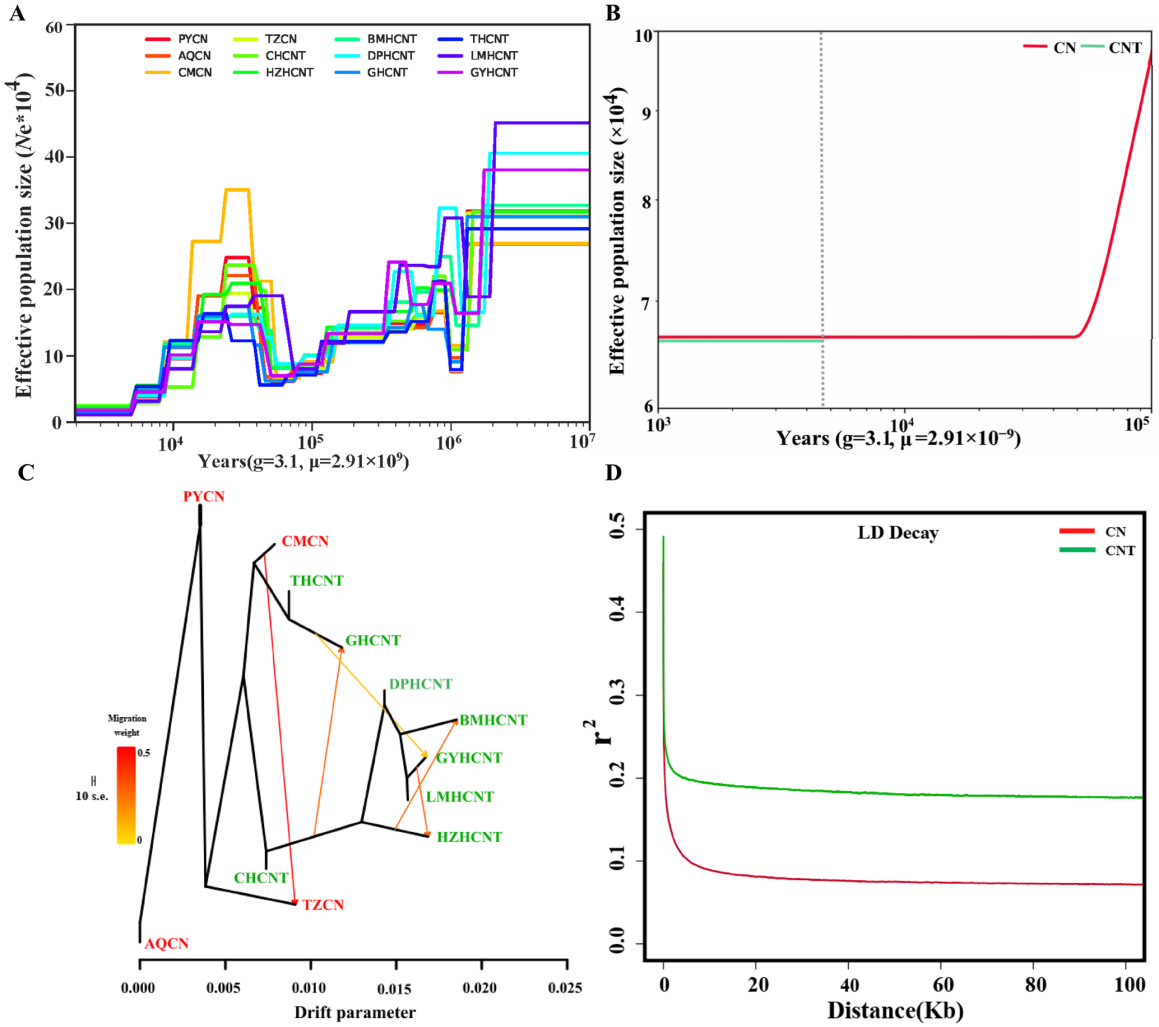
Demographic history and patterns of gene flow between anadromous *C. nasus* and freshwater‐resident *C. nasus taihuensis*. (A) Results from SMC++ analysis illustrate dynamic fluctuations in effective population size (*Ne*) of anadromous *C. nasus* and freshwater‐resident *C. nasus taihuensis*. (B) Estimation of divergence time between anadromous *C. nasus* and freshwater‐resident *C. nasus taihuensis* using SMC++ analysis. The red line represents the inferred ancestral population of the anadromous lineage, and the green line represents that of the freshwater‐resident lineage. The gray dashed line indicates the estimated divergence time of approximately 4.6 thousand years ago (Kya). (C) Maximum likelihood tree of 12 geographic populations with mixture events. Arrows denote the direction and migration weight of gene flow. The scale bar corresponds to the average SE across entries in the sample covariance matrix. (D) Patterns of linkage disequilibrium (LD) decay in anadromous *C. nasus* and freshwater‐resident *C. nasus taihuensis*. The X‐axis represents the physical distance between SNPs, and the Y‐axis shows the *r* ^2^ measure of LD.

We calculated pairwise *F*st values between geographical populations using high‐quality SNPs and subsequently computed the average. The pairwise *F*st values ranged from 0.00001 to 0.1122, with a genome‐wide mean of 0.0448 ± 0.0615, indicating varying levels of genetic differentiation among populations. Among the 12 groups, four anadromous populations (PY, AQ, TZ and CM) exhibited closer genetic relationships with the TH, CH and GH populations (0.00001 < *F*st < 0.0391) than with the HZH, BMH, GYH, LMH and DPH populations (0.0696 < *F*st < 0.1122) (Table S2). The pairwise *F*st values between four anadromous populations and five freshwater‐resident populations (HZH, BMH, GYH, LMH, and DPH) are larger than 0.05 but smaller than 0.15, indicating moderate genetic differentiation. Furthermore, the *F*st values between the anadromous populations and three other freshwater‐resident populations (TH, CH and GH) were all below 0.05, indicating low genetic divergence (Figure 2E). Linkage disequilibrium (LD, measured as *r* ^2^) decayed to half its maximum value at 0.08 kb in anadromous *C. nasus* , compared to 0.4 kb in freshwater‐resident *C. nasus taihuensis* (Figure 3D). LD decay rates varied significantly across the eight freshwater‐resident geographical populations. The TH and CH populations displayed the fastest LD decay (0.3 kb and 0.7 kb, respectively), whereas the LMH population exhibited the slowest decay (68.2 kb), followed by the GYH population (57.8 kb) (Figure S2).

### 3.4 Demographic History and Gene Flow

The demographic history of anadromous *C. nasus* and freshwater‐resident *C. nasus taihuensis* was analyzed to better understand their evolutionary trajectories. Changes in effective population size (*Ne*) were estimated using SMC++ analysis. Both the anadromous and freshwater‐resident populations exhibited similar demographic patterns, including two bottleneck events over the past one million years (Figure 3A). According to the SMC++ results, all populations experienced a notable decline in *Ne* during the period of 4–30 thousand years ago (Kya), with the lowest *Ne* values occurring during the Last Glacial Period. The divergence between anadromous *C. nasus* and freshwater‐resident *C. nasus taihuensis* was estimated to have occurred approximately 4.6 Kya (Figure 3B). To infer potential migration events among the 12 geographical populations, TreeMix analyses were conducted with migration edges ranging from 0 to 10 to investigate possible gene flow patterns. For each predefined number of migration edges, the analysis was performed nine times. The OptM results indicated m = 5 as the optimal number of migration edges (Figure S3). In this model, the strongest migration signal was detected from the branch node representing the CM population to the TZ population of anadromous *C. nasus*. Furthermore, TreeMix analysis identified recent gene flow from five populations (HZH, BMH, GYH, LMH, and DPH) into the GH population. The maximum likelihood (ML) tree corroborated the absence of gene flow between anadromous *C. nasus* and freshwater‐resident *C. nasus taihuensis* (Figure 3C).

### 3.5 Genome‐Wide Selection Signal and Candidate Gene Analysis

**FIGURE 4:**
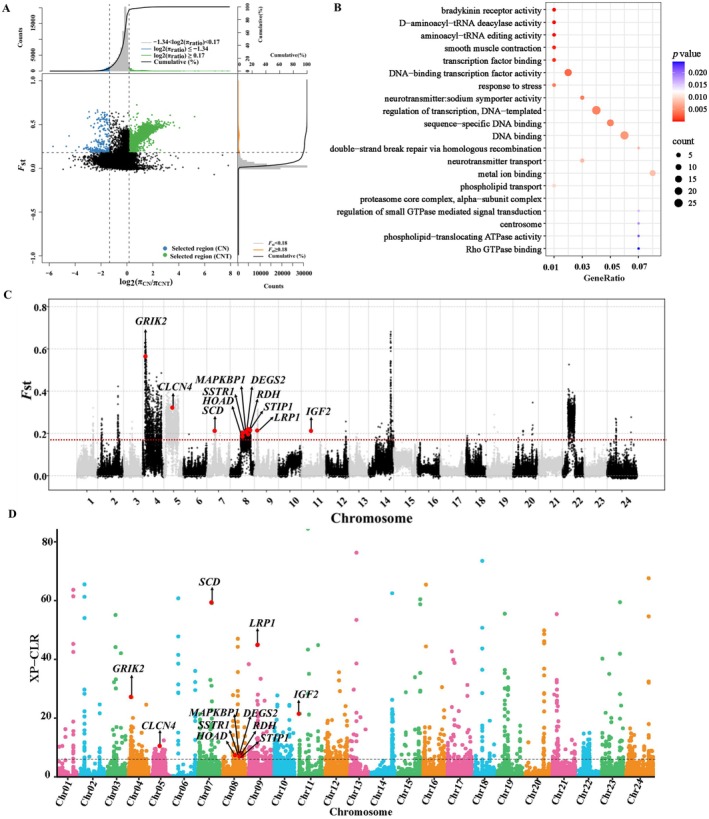
Identification of divergent genomic regions between anadromous *C. nasus* and freshwater‐resident *C. nasus taihuensis*. (A) Distribution of log2 (*π* ratio) and *F*st values across 10 kb sliding windows with a 5 kb step size. In the upper left panel, blue points represent selective sweep regions for anadromous *C. nasus* , defined as those falling within bottom 5% *π* ratio (≤ −1.34) and top 5% *F*st (≥ 0.18). In the upper right panel, green points indicate selective sweep regions for freshwater‐resident *C. nasus taihuensis*, defined as those within the top 5% *π* ratio (≥ 0.17) and top 5% *F*st (≥ 0.18). The vertical and horizontal dashed lines denote the 5% thresholds for *π* ratio (−1.34 and 0.17) and *F*st (0.18), respectively. (B) Top 20 significantly enriched GO terms among candidate genes in anadromous *C. nasus* . (C) Manhattan plot illustrating the genomic distribution of selective signals. Candidate genes potentially associated with migratory adaptation are highlighted in red dots. The dashed line represents the significance threshold for selected regions (*F*st = 0.18). (D) Chromosomal distribution of XP‐CLR scores. Candidate genes potentially associated with migratory adaptation are indicated by red dots. The dashed line represents the significance threshold for selected regions.

We applied an integrative approach (*F*st, *π* ratio, and XP‐CLR) to detect signatures of selective sweeps that contribute to genetic differentiation between anadromous *C. nasus* and freshwater‐resident *C. nasus taihuensis* at the whole‐genome level. Candidate genomic regions were identified based on stringent thresholds: the top 5% of maximum *F*st values (*F*st ≥ 0.18) and *π* ratio thresholds (log_2_(*π*_CNT_/*π*_CN_ ≤ −1.34)), resulting in the detection of 322 candidate genes in anadromous *C. nasus* (Figure 4A). To further detect genomic regions with extreme allele frequency differentiation, XP‐CLR scores were calculated across the genome. Windows exceeding the top 5% threshold (XP‐CLR > 5.74) were considered as selection regions, yielding 2108 candidate genes (Figure 4D). Among them, a total of 290 genes identified by all three metrics were found to overlap and were considered candidate genes of anadromous *C. nasus* . These candidate genes were significantly enriched in GO terms (*p* < 0.05) closely related to smooth muscle contraction (GO: 0006939), neurotransmitter: sodium symporter activity (GO: 0005328), phospholipid transport (GO: 0015914) and DNA‐binding transcription factor activity (GO: 0003700) (Figure 4B and Table S3). These genes were notably enriched in energy metabolism, including *SCD*, *HOAD* and *DEGS2*. Anadromous *C. nasus* generally intake few foods during spawning migration and primarily depends on stored energy reserves. The selected genes may play a role in the metabolic and transport processes of energy substrates. Moreover, enrichment analysis revealed that several genes associated with osmoregulation have undergone positive selection, enabling this euryhaline fish to adapt to fluctuating salinity levels (Table S4). Key candidate genes, such as *CLCN4*, *MAPKBP1*, *STIP1*, *IGF2*, *GRIK2* and *LRP1*, were identified as playing crucial roles in maintaining physiological balance under varying osmotic conditions (Figure 4C and Table 2).

**TABLE 2:** Several genes exhibiting selection signal in *C. nasus* and *C. nasus taihuensis* were significantly enriched in pathways associated with lipid metabolism and osmoregulation (*p*‐value < 0.05). These candidate genes were identified through an integrative approach (*F*st, *π* ratio, and XP‐CLR), all of which met the selection threshold when comparing anadromous and freshwater‐resident ecotypes. The chromosome ID indicates the specific chromosome on which each gene is located, while the Gene ID reflects the approximate physical position of the gene based on the reference genome assembly of *C. nasus* .

Ecotype	Chromosome ID	Gene ID	Gene name	Description	Gene function	*p*
*C. nasus*	Chr07	G04963	*SCD*	stearoyl‐CoA desaturase (Delta‐9 desaturase)	Lipid metabolism	0.03
	Chr08	G05722	*HOAD*	long‐chain 3‐hydroxyacyl‐CoA dehydrogenase	Lipid metabolism	0.02
	Chr08	G05898	*DEGS2*	sphingolipid 4‐desaturase	Lipid metabolism	0.02
	Chr08	G05864	*SSTR1*	somatostatin receptor 1	Hormonal regulation	0.03
	Chr05	G03309	*CLCN4*	chloride intracellular channel protein 4	Osmoregulation	0.03
	Chr08	G05878	*MAPKBP1*	mitogen‐activated protein kinase binding protein 1	Osmoregulation	0.04
	Chr08	G06029	*STIP1*	stress‐induced‐phosphoprotein 1	Osmoregulation	0.01
	Chr11	G01625	*IGF2*	insulin‐like growth factor 2	Osmoregulation	0.02
	Chr04	G02821	*GRIK2*	glutamate receptor ionotropic	Osmoregulation	0.03
	Chr09	G06304	*LRP1*	low‐density lipoprotein receptor‐related protein 1	Osmoregulation	0.04
*C. nasus taihuensis*	Chr04	G02657	*ATP1A1*	sodium/potassium‐transporting ATPase subunit alpha	Osmoregulation	0.00
	Chr05	G03023	*AQP9*	aquaporin‐9	Osmoregulation	0.03
	Chr04	G02261	*NBC*(SLC4A4A)	solute carrier family 4 (sodium bicarbonate cotransporter)	Osmoregulation	0.01
	Chr05	G03449	*NHE3* (SLC9A6)	solute carrier family 9 (sodium/hydrogen exchanger), member 6	Osmoregulation	0.03
	Chr05	G03023	*PLEC*	Plectin	Osmoregulation	0.03
	Chr04	G02267	*TGM4*	transglutaminase 4	Osmoregulation	0.03

Meanwhile, 714 candidate genes were detected when freshwater‐resident *C. nasus taihuensis* was compared to anadromous *C. nasus* (euryhaline fish). Selection on these genes may underlie the genetic basis for different ecotypes of *C. nasus* to adapt to heterogeneous environments. Functional annotation was performed to explore the biological functions of these candidate genes. For freshwater‐resident *C. nasus taihuensis*, the most significantly enriched terms included ion transport, ion channel activity, ion transmembrane transport, MAPK signaling pathway (*p* < 0.05). We found that seven candidate genes on Chr04 and Chr05 functioned crucially in osmotic homeostasis, including *ATP1A1*, *TGM4*, *NBC*, *AQP9*, *NHE3*, *PLEC*, and *CA4* (Table 2, Figure S4, and Table S5).

## 4 Discussion

### 4.1 Evolutionary Patterns of Two Ecotypes *C. nasus*

The taxonomic status of two ecotypes ( *C. nasus* and *C. nasus taihuensis*) within the *C. nasus* species complex has long been unsolved, as previous morphological and molecular evidence has yielded conflicting classifications. Two controversial taxonomic treatments have emerged: (i) both *C. nasus* and *C. nasus taihuensis* were a single species, or (ii) *C. nasus taihuensis* was treated as a distinct subspecies within *C. nasus* (Gao et al. 2021). However, these conclusions were reached based on limited morphological characteristics and traditional molecular markers, which were insufficient to clarify the evolutionary relationships within this species complex. Based on multiple analyses (NJ tree, PCA and ADMIXTURE analysis), our study confirmed that *C. nasus taihuensis* is not a valid subspecies but rather an ecotype of *C. nasus* , which validated the first taxonomic hypothesis at the population genomic level. In particular, the close genetic relationships between *C. nasus* and *C. nasus taihuensis* were supported by low differentiation index (average *F*st = 0.0448 < 0.05). Moreover, recent studies using microsatellite markers or few SNP loci have consistently reported low levels of genetic divergence between the two forms, supporting that *C. nasus taihuensis* represents an ecotype of migratory *C. nasus* (Cheng et al. 2019; Xuan et al. 2021).

During the Last Glacial Maximum (LGM), approximately 18,000 years ago, the Yangtze River became an inland river, isolated from the East China Sea due to arid climate, reduced rainfall, and desertification (Xiao et al. 2003). As a result, anadromous *C. nasus* populations were restricted to the main deep‐water channels of the Yangtze River during this glacial period, promoting the formation of freshwater resident populations through geographic isolation (Xue et al. 2019). Approximately 7000 years ago, rising sea levels rapidly filled the modern lakes in the lower reaches of the Yangtze River (Chen and Stanley 1998). By around 6000 years ago, ongoing sea‐level fluctuations caused a significant rise in the water level of Taihu Lake (Wang et al. 2001). Freshwater populations originating from the Yangtze River subsequently colonized Taihu Lake. The divergence time estimated using the SMC++ method indicated that freshwater‐resident populations split from their ancestral lineage approximately 4.6 Kya, a timeframe that aligns closely with the geological formation of Taihu Lake. Over the past millennium, additional lakes, including Gehu Lake and Chaohu Lake, formed successively in the region (Wang and Chen 1999). *C. nasus taihuensis* gradually expanded into these emerging lakes and became the dominant species within these lacustrine ecosystems. However, since the 1950s, these lakes have been disconnected from rivers due to the construction of dike or sluice gates (Ru and Liu 2013; Liu and Wang 2010). For instance, Taihu Lake has lost its natural hydrological connection with the Yangtze River (Qin et al. 2010), while Dongping Lake can no longer exchange water freely with the Yellow River (Song et al. 2012). The loss of river–lake connectivity has restricted the free movement of *C. nasus taihuensis*. In our study, TreeMix analysis revealed no evidence of gene flow between the two ecotypes of *C. nasus* , suggesting that geographic isolation between lakes and rivers limits gene flow. In the absence of gene flow, divergent ecological selection is supposed to maintain genetic differentiation.

Anadromous *C. nasus* is an economically important fish species in China due to its high nutritional value and cultural significance. However, wild populations of *C. nasus* have declined sharply by 98% between 1973 and 2012, a reduction primarily attributed to human activities such as overfishing and habitat degradation (Shen et al. 2015). In contrast, freshwater‐resident *C. nasus taihuensis* has relatively lower commercial value (Liu et al. 2014). Population genomic analyses based on high‐depth sequencing data reveal that anadromous *C. nasus* exhibit lower genetic diversity than freshwater‐resident *C. nasus taihuensis* (0.0021 vs. 0.0027). Previous studies have shown that wild populations of *C. nasus* in Poyang Lake display reduced genetic diversity and signs of germplasm degradation (Zhang et al. 2023). Our findings suggest that anthropogenic factors have likely driven a more pronounced decline in anadromous populations compared to their freshwater counterparts in recent decades. Consequently, the conservation of *C. nasus* germplasm resources has become increasingly urgent.

### 4.2 Signatures of Adaptive Divergence of *C. nasus*

Exploring genetic variations underlying adaptive traits can provide valuable resources for effective conservation and management of *C. nasus* germplasm. Here, we compared the genomes of anadromous and freshwater‐resident populations to identify signatures of positive selection under adaptive divergence. A total of 290 overlapping genes were identified as potential genes associated with adaptation to long‐distance migration. Migratory individuals migrate hundreds of kilometers from the Yangtze River estuary to their natal spawning grounds, during which they rarely feed and must primarily rely on stored energy reserves (Ma et al. 2020). Under conditions of prolonged food deprivation, lipid reserves serve as a critical and readily mobilizable energy source. We identified strong signatures of selection in genes associated with lipid metabolism in anadromous *C. nasus* , including *SSTR1*, *SCD*, *HOAD* and *DEGS2*. Specifically, stearoyl‐CoA desaturase (*SCD*), the rate‐limiting enzyme in biosynthesis of monounsaturated fatty acids (MUFAs), introduced the first double bond at the Δ9 position of saturated fatty acids (SFA), thereby converting them into MUFAs (Cohen et al. 2002). During migration, there is a rapid development of sexual maturity, and the synthesized unsaturated fatty acids were transferred into the gonads, influencing both the quantity and quality of eggs in *C. nasus* (Izquierdo et al. 2001). *HOAD* serves as a key regulatory enzyme in the β‐oxidation pathway, which is responsible for catabolism of free fatty acids (FFAs) (Guglielmo et al. 2002). A proportion of FFA, after being converted into acetyl‐CoA through β‐oxidation, enters the Krebs cycle and electron transport chain for complete oxidation, thereby supplying the energy required for spawning migration of *C. nasus* . The *SSTR1* gene encodes a receptor for somatostatin, a hormone essential for regulating the balance between energy allocation for migration and oocyte development (Sheridan and Kittilson 2004). Similar results have been reported in steelhead/rainbow trout ( *Oncorhynchus mykiss* ) (Poppinga et al. 2007). These selected genes associated with lipid metabolism were found in our study, which could contribute to highly efficient long‐distance migration. Additionally, we found a few genes (*CLCN4*, *MAPKBP1*, *STIP1*, *IGF2*, *GRIK2* and *LRP1*) with strong signals of selection in anadromous *C. nasus* , which were functionally related to osmoregulation. For instance, insulin‐like growth factor 2 (*IGF2*), which is crucial for seawater adaptation (Mancera and McCormick 1998), may be functionally conserved in anadromous *C. nasus* . Similarly, *Igf* has been shown to promote salinity tolerance in Atlantic salmon, with elevated plasma levels of *Igf* (McCormick 2001).

*C. nasus taihuensis* have adapted locally through long‐term selection under freshwater environments. *C. nasus taihuensis* remains confined to freshwater lakes, such as Hongze Lake, Gaoyou Lake, Luoma Lake, and Dongping Lake, and has adapted to survive without migration. We found a few genes (*ATP1A1*, *AQP9*, *CA*, and *NHE3*) involved in ion transport and water channel functions were significantly selected in *C. nasus taihuensis*, which are critical for maintaining osmotic and ionic balance (Cutler and Cramb 2001). *ATP1A1* encodes an ion transport protein, exhibiting significant genetic differentiation between marine and freshwater sticklebacks (DeFaveri et al. 2011; Jones et al. 2012). ATPase plays a crucial role in maintaining ion balance and electrolyte homeostasis across various osmoregulatory epithelia, supporting its adaptive significance in ion uptake for freshwater *C. nasus taihuensis* (Evans et al. 2005; Sutherland et al. 2014). Aquaporins (*AQPs*) facilitate rapid osmoregulation by enabling the diffusion of water and osmolytes across cellular membranes (Hill et al. 2004). In *Lateolabrax maculatus*, the *AQPs* gene has been associated with water metabolism and osmotic pressure regulation (Zhang et al. 2017). Our study showed that *AQP9* may regulate the exchange of water molecules between intracellular and extracellular environments (Hill et al. 2004). Additionally, we examined other genes potentially involved in freshwater osmoregulation, such as carbonic anhydrases (*CA*). In zebrafish, *CA* promotes epithelial Na^+^ and Cl^−^ uptake as well as H^+^ secretion under freshwater conditions (Ito et al. 2013). In steelhead trout ( *Oncorhynchus mykiss* ), a genome‐wide scan has identified selective sweep signals in the *CA* gene following the transition from a marine to a freshwater lake environment (Willoughby et al. 2018). It is plausible that the *CA* gene exerts comparable functional effects in *C. nasus taihuensis*, potentially playing a role in the regulation of osmotic pressure. These selected genes may contribute to the divergence between *C. nasus* and *C. nasus taihuensis*.

## Author Contributions

**Fengjiao Ma:** data curation (equal), methodology (equal), visualization (equal), writing – original draft (equal). **Sheng Wang:** methodology (equal). **Wenzhi Ma:** methodology (equal), visualization (equal). **Hui Wang:** investigation (equal), methodology (equal). **Haotian Jin:** formal analysis (equal), methodology (equal), visualization (equal). **Legen Peng:** methodology (equal), visualization (equal). **Guojun Yin:** project administration (equal), supervision (equal), writing – review and editing (equal). **Kai Liu:** funding acquisition (equal), supervision (equal), writing – review and editing (equal).

## Conflicts of Interest

The authors declare no conflicts of interest.

## Supporting information


**Data S1:** ece372815‐sup‐0001‐DataS1.docx.

**Data S2:** ece372815‐sup‐0002‐DataS2.docx.

**Figure S1:** Panoramas of the distribution of SNPs across 24 chromosomes of *Coilia nasus* . Chromosome length is displayed on the x‐axis, with each band representing a chromosome. The genome is divided into 100 kb segments, and each segment is colored based on the number of SNPs. Darker areas indicate regions where SNPs are concentrated.
**Figure S2:** LD decay patterns of eight populations of freshwater‐resident *C. nasus taihuensis*.
**Figure S3:** The output generated by OptM for the simulated dataset containing *m* = 5 migration edges. (a) The mean and standard deviation (SD) across 10 iterations for the composite likelihood L (*m*), represented by black circles on the left axis, and the proportion of variance explained, indicated by red “x” markers on the right axis. (b) The second‐order change rate (Δ*m*) across different values of *m*.
**Figure S4:** GO enrichment for the selected genes of freshwater‐resident *C. nasus taihuensis* based on *F*st values and *π* ratio.
**Table S1:** Statistics of reads mapping and coverage of 128 samples of *Coilia nasus* used in population genomics.
**Table S2:** The genetic differentiation index (*F*st) among the 12 geographical populations of *Coilia nasus* covering the Yangtze River, Huaihe River and Yellow River system.
**Table S3:** Significantly enriched Gene Ontology (GO) terms of candidate genes exhibiting selection signals in anadromous *Coilia nasus*.
**Table S4:** Significantly enriched KEGG pathways of candidate genes exhibiting selection signals in anadromous *Coilia nasus*.
**Table S5:** Significantly enriched Gene Ontology (GO) terms candidate genes exhibiting selection signals in freshwater‐resident *Coilia nasus taihuensis*.

## Data Availability

The whole‐genome sequence clean reads for *C. nasus* were deposited in the NCBI Sequence Read Archive (SRA) under accession no. PRJNA1193256. The codes and scripts of data filtering, calling SNPs, population genetic analysis, and selected regions analysis are available in the Data S1 and S2.
