# Supplementary material for: Population Genomics Provides Novel Insights Into Evolutionary Relationships and Local Adaptation of Two Ecotypes Coilia nasus
Source: Ecol Evol. 2025 Dec 23;15(12):e72815. doi: 10.1002/ece3.72815 (PMC12723445; doi:10.1002/ece3.72815)
Supplement: Supplementary file 1 — Data S1: ece372815‐sup‐0001‐DataS1.docx. [file ECE3-15-e72815-s001.docx]

##01. Set the reference genome and software paths

REF=ref_genome.fa

sample=AQCN01

GATK=~/gatk-4.1.2.0/gatk

PLINK=~/plink

VCFTOOLS=~/vcftools

bcftools=~/bcftools

##02. Call SNPs

#Run HaplotypeCaller in parallel for each sample

java -Xmx4G -Djava.io.tmpdir=./Tmp/${sample}/ $GATK HaplotypeCaller -R $REF_FA -I $BAM --standard-min-confidence-threshold-for-calling 20 -native-pair-hmm-threads 1 --output-mode EMIT_ALL_SITES -ERC GVCF -O ${sample}.gvcf.gz

# merge all sample gvcf to gvcf

gvcf_var=$(for i in `ls -d *`; do echo -e "--variant ${sample}.gvcf.gz \c"; done)

java -Xmx32G -Djava.io.tmpdir=./Tmp/ -jar $GATK4 CombineGVCFs -R $REF $gvcf_var -O All.gvcf.gz

# gvcf to vcf

java -Xmx32G -Djava.io.tmpdir=./Tmp/ -jar $GATK4 GenotypeGVCFs -R $REF --include-non-variant-sites true -V All.gvcf.gz -O All.raw.vcf.gz

# filter snps

java -Xmx32G -Djava.io.tmpdir=./Tmp/ -jar $GATK4 SelectVariants -R $REF -V All.raw.vcf.gz --select-type-to-include SNP -O All.snp.vcf.gz

java -Xmx32G -Djava.io.tmpdir=./Tmp/ -jar $GATK4 VariantFiltration -R $REF -V All.snp.vcf.gz -O All.filter_snp.vcf.gz --filter-name "SNP_filter" --filter-expression "QD < 2.0 || MQ < 40.0 || FS > 60.0 || SOR > 3.0 || MQRankSum < -12.5 || ReadPosRankSum < -8.0"

$VCFTOOLS --gzvcf All.filter_snp.s1.vcf.gz --max-missing 0.9 --maf 0.05 --min-meanDP 5 --min-alleles 2 --max-alleles 2 --recode --recode-INFO-all --stdout | $bcftools view -Oz -o END.vcf.gz;

##03. Population genetic analysis

# split total vcf to group vcf

for Breed in ${Group_List[@]};

do

$bcftools view -S $Breed.list -m2 -M2 END.vcf.gz -Oz -o $Breed.pre_filter.vcf.gz;

$VCFTOOLS --gzvcf $Breed.pre_filter.vcf.gz --min-meanDP 5 --max-missing 0.9 --maf 0.05 --min-alleles 2 --max-alleles 2 --recode --recode-INFO-all --out ./00.Group/$Breed;

mv ./00.Group/$Breed.recode.vcf ./00.Group/$Breed.vcf;

gzip ./00.Group/$Breed.vcf;

$bcftools index ./00.Group/$Breed.vcf.gz;

done

# 1) PI

for Breed in ${Group_List[@]};

do

$VCFTOOLS --gzvcf ./00.Group/$Breed.vcf.gz --window-pi 40000 --out ./01.PI/$Breed.window-pi

done

# 2) FST

cd ./02.FST;

Group_List_len=${#Group_List[@]};

for ((i=0;i<=(Group_List_len-1);i++))

do

for((j=i+1;j<=(Group_List_len-1);j++))

do

$VCFTOOLS --gzvcf ./00.Group/END.vcf.gz --fst-window-size 40000 --weir-fst-pop ./00.Group/${Group_List[$i]}.list --weir-fst-pop ./00.Group/${Group_List[$j]}.list --out ./02.FST/${Group_List[$i]}.VS.${Group_List[$j]}.fst;

done

done

# 3)Tajima'D

cd ./03.TajimaD;

$VCFTOOLS --gzvcf ./00.Group/END.vcf.gz --TajimaD 40000

for Breed in ${Group_List[@]};

do

$VCFTOOLS --gzvcf ./00.Group/$Breed.vcf.gz --TajimaD 40000 --out ./03.TajimaD/$Breed.window-tajimaD

done

# 4)# LD

for Breed in ${Group_List[@]}

do

$PopLDdecay -InVCF $Breed.vcf.gz -OutStat $Breed.Lddecay

done

# PCA

$PLINK --vcf END.vcf.gz --make-bed --dog --out SNP.ID.CHROM

/gcta_v1.94.0beta_linux/gcta64 --make-grm --bfile plink --autosome-num 100 --out plink.gcta ;

/gcta_v1.94.0beta_linux/gcta64 --grm plink.gcta --pca 20 --out plink.gcta;

# structure

for K in 2 3 4 5 6

do admixture --cv SNP.ID.CHROM.bed $K | tee log${K}.out; done

# phylogenetic tree

~/VCF2Dis-1.47/bin/VCF2Dis -InPut END.vcf.gz -OutPut 01.p_dis.mat;

~/PHYLIPNEW-3.69.650/bin/fneighbor -datafile 01.p_dis.mat -outfile 02.tree.out -matrixtype s -treetype n -outtreefile 03.tree.out.tre;
