# Supplementary material for: Population Genomics Provides Novel Insights Into Evolutionary Relationships and Local Adaptation of Two Ecotypes Coilia nasus
Source: Ecol Evol. 2025 Dec 23;15(12):e72815. doi: 10.1002/ece3.72815 (PMC12723445; doi:10.1002/ece3.72815)
Supplement: Supplementary file 2 — Data S2: ece372815‐sup‐0002‐DataS2.docx. [file ECE3-15-e72815-s003.docx]

library (RColorBrewer)

#mycol<-brewer.pal(8,"Set1")

mycol<-c("black","red","blue","green")

filein<-"HRCL-vs-DPL.0.05.all"

palette(mycol)

data<-read.table(filein,sep="\t",header=F)

win_total<-nrow(data)

pdf("HRCL-vs-DPL.0.05.DPL.selected.region.pi_fst.pdf",h=10,w=10)

layout(matrix(c(2,0,1,3),2,2,byrow=TRUE), widths=c(3,1),heights=c(1,3), TRUE)

#-----------------------------------------------------------------------------------

###the first#

par(mar=c(5.1,5.1,0.1,0))

for(i in 1:length(data[,8]))

{ if(data[i,8]<0)

data[i,8]=0

}

plot(data[,8]~data[,7],pch=19,col=as.integer(data[,dim(data)[2]]),xlab="log2(Pi ratio(PiHRCL/PiDPL))",ylab="Fst",cex.lab=1.5,cex.axis=1.5,cex=0.5)

legend("topleft",legend="DPL selected region",pch=19,col=2,bty="n",cex=2)

abline(h=0.572101,col="black",lty=2,lwd=2)

abline(v=0.52771185631076,col="black",lty=2,lwd=2)

#-----------------------------------------------------------------------------------

###the second

par(mar=c(0,5.1,3,0))

pi_hist<-hist(data[,7],breaks=seq(from=min(data[,7]),to=max(data[,7]),length.out=120),plot=FALSE)

pi_hist$counts2<-pi_hist$counts*100/sum(pi_hist$counts)

hist(data[,7],breaks=seq(from=min(data[,7]),to=max(data[,7]),length.out=120),ann=FALSE,axes=T,xaxt="n",yaxt="n",xlab='', ylab='',col=3,border=3,main="Pi density")

y_pi_pos<-seq(0,max(pi_hist$counts),length.out=6)

y_pi_label<-round(y_pi_pos*100/sum(pi_hist$counts),digits=0)

y_pi_pos<-y_pi_label*dim(data)[1]/100

axis(side=2, y_pi_pos,labels=FALSE)

mtext(y_pi_label,side=2,las=1,at=y_pi_pos, line=0.8, cex=1.2)

mtext("Frequence (%)",side=2, line=3, at=max(pi_hist$counts)/2,cex=1.2)

abline(v=0.52771185631076,col="black",lty=2,lwd=2)

par(new=T,ann=F)

par(mar=c(0,4.1,3,0))

num<-length(unique(data[,7]))

pi_cum<-array(0:0,c(num,2))

j=1

for(i in sort(unique(data[,7]))){

pi_cum[j,1]=i

pi_cum[j,2]=(colSums(data[,c(7,8)]<=i)[1])*100/dim(data)[1]

j=j+1

}

plot(pi_cum[,1],pi_cum[,2],type='l', lwd=1, bty='n',xaxt='n',yaxt='n', xlab='', ylab='', ylim=c(0, 100))

y_pi_cum_pos <- seq(0,100,by=20)

axis(side=4, y_pi_cum_pos,labels=FALSE)

mtext(y_pi_cum_pos,side=4,las=1,at=y_pi_cum_pos, line=0.8, cex=1.2)

mtext('Cumulative (%)',side=4, line=3, at=median(y_pi_cum_pos)+10, cex=1.2)

par(mar=c(5.1,0.2,0.1,1))

num<-length(unique(data[,8]))

fst_cum<-array(0:0,c(num,2))

j=1

for(i in sort(unique(data[,8]))){

fst_cum[j,1]=i

fst_cum[j,2]=(colSums(data[,c(7,8)]<=i)[2])*100/dim(data)[1]

j=j+1

}

plot(fst_cum[,2],fst_cum[,1],type='l', lwd=1, bty='n',xaxt='n',yaxt='n', xlab='', ylab='', ylim=c(min(data[,8]),max(data[,8])))

y_fst_cum_pos <- seq(0,100,by=20)

axis(side=3, y_fst_cum_pos,labels=FALSE)

mtext(y_fst_cum_pos,side=3,las=1,at=y_fst_cum_pos, line=0.8, cex=1.2)

mtext('Cumulative (%)',side=3, line=3, at=median(y_fst_cum_pos)+10, cex=1.2)

abline(h=0.572101,col="black",lty=2,lwd=2)

par(new=T,ann=F)

yhist <- hist(data[,8],breaks=seq(from=min(data[,8]),to=max(data[,8]),length.out=100),plot=FALSE)

par(mar=c(5.1,0.35,0.1,1))

#barplot(yhist$density,horiz=TRUE,space=0,axes=T,col=rgb(0,1,0,alpha=0.5),main="",cex.axis=1.5,border=rgb(0,1,0,alpha=0.5))

barplot(yhist$density,horiz=TRUE,space=0,xaxt="n",yaxt="n",col=rgb(0,1,0,alpha=0.5),main="",cex.axis=1.5,border=rgb(0,1,0,alpha=0.5))

x_fst_pos<-seq(0,max(yhist$density),length.out=6)

x_fst_label<-round(x_fst_pos*100/sum(yhist$density),digits=0)

axis(side=1,labels=x_fst_label,at=x_fst_pos)

mtext("Frequence (%)",side=1, line=3, at=max(yhist$density)/2,cex=1.2)

dev.off()

q(save="no")
