# Supplementary material for: Population Genomics Provides Novel Insights Into Evolutionary Relationships and Local Adaptation of Two Ecotypes Coilia nasus
Source: Ecol Evol. 2025 Dec 23;15(12):e72815. doi: 10.1002/ece3.72815 (PMC12723445; doi:10.1002/ece3.72815)
Supplement: Supplementary file 3 — Figure S1: Panoramas of the distribution of SNPs across 24 chromosomes of Coilia nasus . Chromosome length is displayed on the x‐axis, with each band representing a chromosome. The genome is divided into 100 kb segments, and each segment is colored based on the number of SNPs. Darker areas indicate regions where SNPs are concentrated. Figure S2: LD decay patterns of eight populations of freshwater‐resident C. nasus taihuensis. Figure S3: The output generated by OptM for the simulated dataset containing m = 5 migration edges. (a) The mean and standard deviation (SD) across 10 iterations for the composite likelihood L (m), represented by black circles on the left axis, and the proportion of variance explained, indicated by red “x” markers on the right axis. (b) The second‐order change rate (Δm) across different values of m. Figure S4: GO enrichment for the selected genes of freshwater‐resident C. nasus taihuensis based on Fst values and π ratio. Table S1: Statistics of reads mapping and coverage of 128 samples of Coilia nasus used in population genomics. Table S2: The genetic differentiation index (Fst) among the 12 geographical populations of Coilia nasus covering the Yangtze River, Huaihe River and Yellow River system. Table S3: Significantly enriched Gene Ontology (GO) terms of candidate genes exhibiting selection signals in anadromous Coilia nasus. Table S4: Significantly enriched KEGG pathways of candidate genes exhibiting selection signals in anadromous Coilia nasus. Table S5: Significantly enriched Gene Ontology (GO) terms candidate genes exhibiting selection signals in freshwater‐resident Coilia nasus taihuensis. [file ECE3-15-e72815-s002.docx]

**Appendix S1**


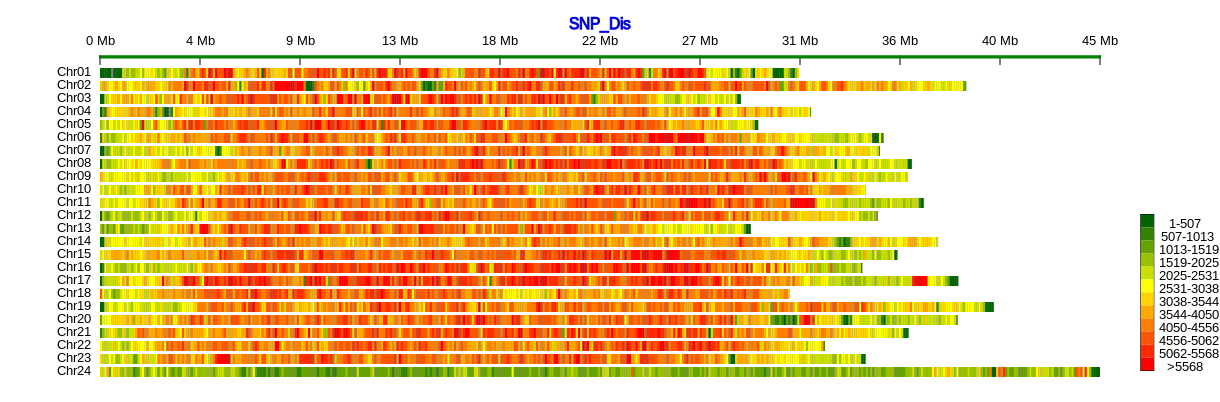


**Figure S1.** Panoramas of the distribution of SNPs across 24 chromosomes of *Coilia nasus*. Chromosome length is displayed on the x-axis, with each band representing a chromosome. The genome is divided into 100 kb segments, and each segment is colored based on the number of SNPs. Darker areas indicate regions where SNPs are concentrated.


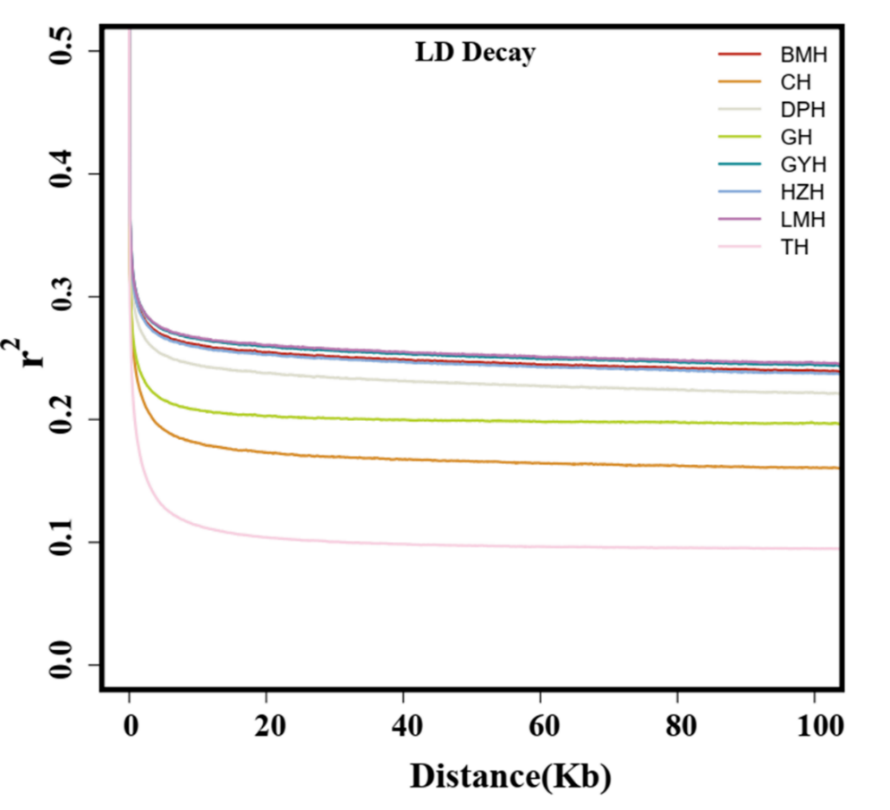


**Figure S2.** LD decay patterns of eight populations of freshwater-resident *C. nasus taihuensis*.


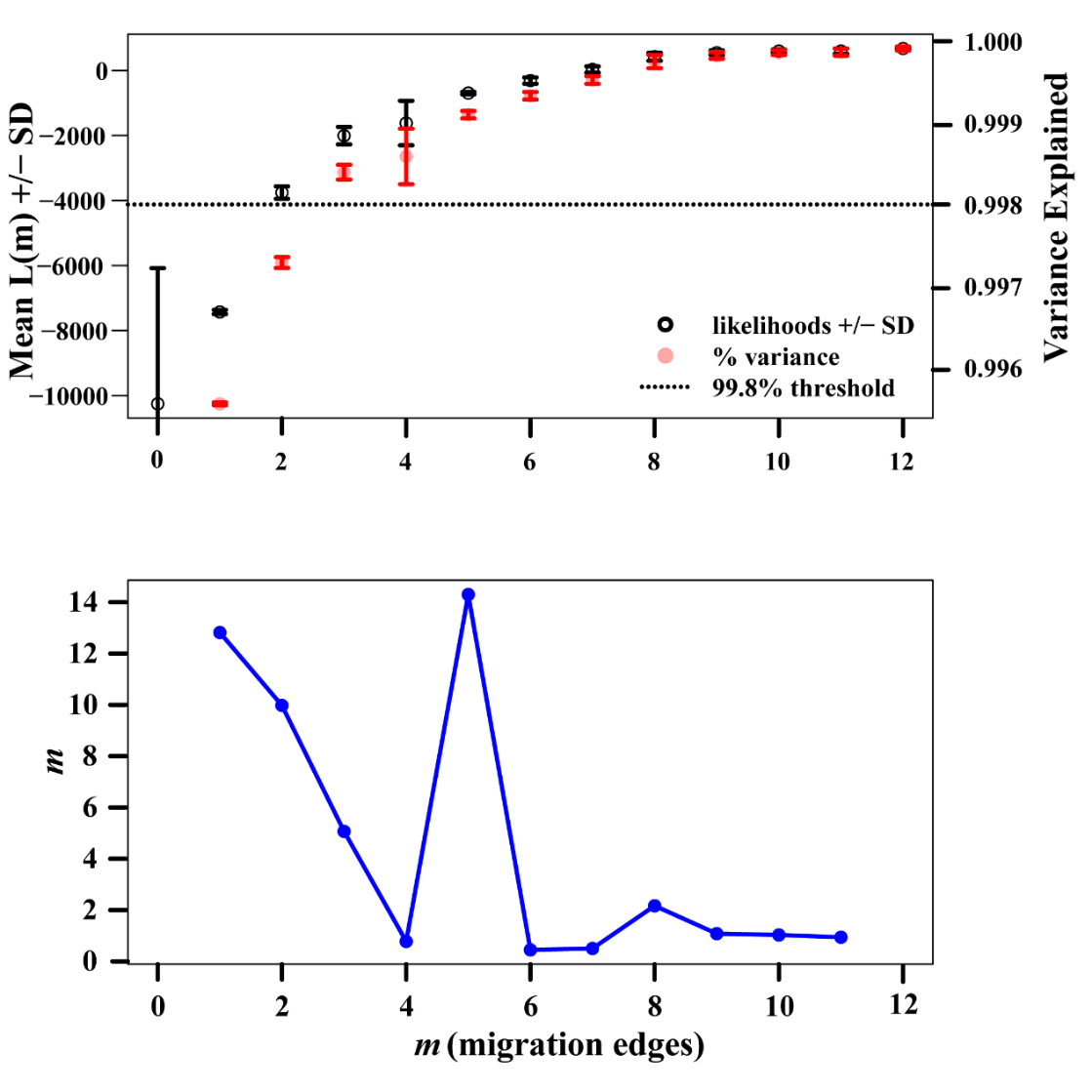


**(b)**

**(a)**

**Figure S3.** The output generated by OptM for the simulated dataset containing *m* = 5 migration edges. (a) The mean and standard deviation (SD) across 10 iterations for the composite likelihood L(*m*), represented by black circles on the left axis, and the proportion of variance explained, indicated by red "x" markers on the right axis. (b) The second-order change rate (Δ*m*) across different values of *m*.


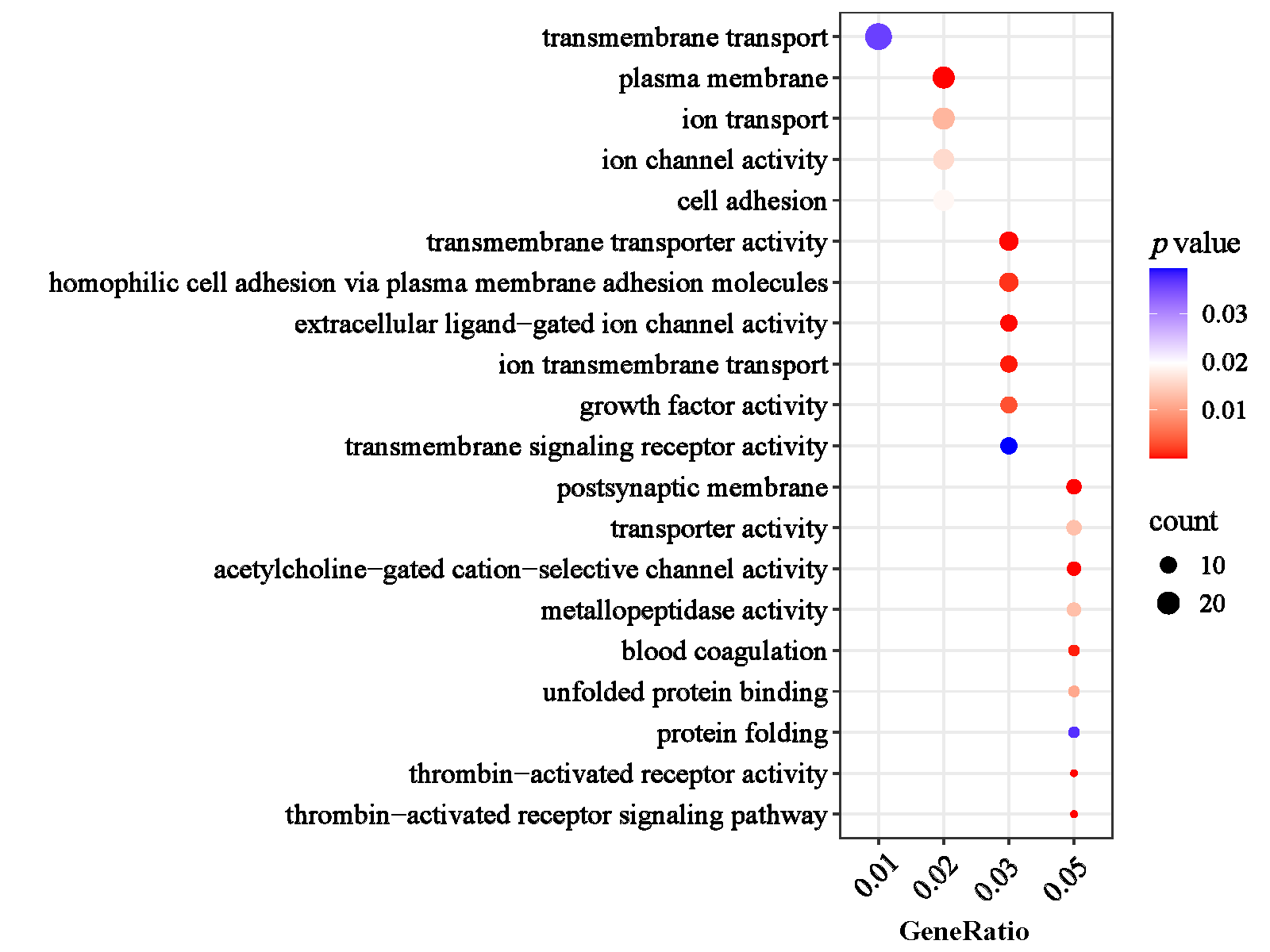


**Figure S4.** GO enrichment for the selected genes of freshwater-resident *C. nasus taihuensis* based on *F*st values and π ratio.

| **Table S1. Statistics of reads mapping and coverage of 128 samples of *Coilia nasus* used in population genomics.** | | | | | |
| --- | --- | --- | --- | --- | --- |
| Group | Sample ID | Clean_Data(G) | Coverage% | Mean Depth | Mapping rate |
| *Coilia nasus* | CMCN01 | 25.34 | 89.33 | 25.38 | 98.34% |
|  | CMCN02 | 26.88 | 89.93 | 27.05 | 98.52% |
|  | CMCN03 | 27.32 | 89.72 | 27.36 | 98.43% |
|  | CMCN04 | 21.55 | 89.02 | 21.65 | 98.26% |
|  | CMCN05 | 28.19 | 91.62 | 28.22 | 98.44% |
|  | CMCN06 | 27 | 91.1 | 27.12 | 98.39% |
|  | CMCN07 | 26.91 | 89.61 | 27.12 | 98.31% |
|  | CMCN08 | 21.36 | 88.44 | 21.4 | 98.45% |
|  | CMCN09 | 25.97 | 91.03 | 25.9 | 98.31% |
|  | CMCN10 | 21.33 | 89.68 | 21.27 | 98.43% |
|  | TZCN01 | 25.27 | 89.74 | 25.54 | 98.41% |
|  | TZCN02 | 28.63 | 90.18 | 28.81 | 98.27% |
|  | TZCN03 | 21.87 | 89.38 | 21.92 | 98.43% |
|  | TZCN04 | 23.45 | 89.32 | 23.58 | 98.44% |
|  | TZCN05 | 26.19 | 89.53 | 26.38 | 98.47% |
|  | TZCN06 | 22.4 | 89.58 | 22.51 | 98.43% |
|  | TZCN07 | 27.56 | 91.3 | 27.63 | 98.32% |
|  | TZCN08 | 34.23 | 91.06 | 34.07 | 98.94% |
|  | TZCN09 | 30.96 | 90.1 | 31.06 | 98.47% |
|  | TZCN10 | 27.98 | 89.74 | 28.1 | 98.49% |
|  | AQCN01 | 23.72 | 89.22 | 23.98 | 98.46% |
|  | AQCN02 | 26.46 | 91.29 | 26.7 | 98.37% |
|  | AQCN03 | 23.73 | 89.54 | 23.91 | 98.43% |
|  | AQCN04 | 26.16 | 89.64 | 26.39 | 98.37% |
|  | AQCN05 | 24.52 | 89.81 | 24.79 | 98.38% |
|  | AQCN06 | 22.48 | 89.07 | 22.78 | 98.26% |
|  | AQCN07 | 21.63 | 89.48 | 21.87 | 98.30% |
|  | AQCN08 | 26.98 | 90.02 | 27.25 | 98.46% |
|  | AQCN09 | 22.59 | 89.65 | 22.97 | 98.47% |
|  | AQCN10 | 25.35 | 89.61 | 25.6 | 98.45% |
|  | PYCN01 | 25.44 | 91.77 | 25.78 | 98.39% |
|  | PYCN02 | 24.96 | 89.64 | 25.3 | 98.57% |
|  | PYCN03 | 23.5 | 89.88 | 23.75 | 98.45% |
|  | PYCN04 | 28.09 | 91.55 | 28.47 | 98.40% |
|  | PYCN05 | 22.59 | 89.5 | 22.85 | 98.47% |
|  | PYCN06 | 23.24 | 89.44 | 23.51 | 98.62% |
|  | PYCN07 | 19.98 | 90.27 | 20.16 | 98.54% |
|  | PYCN08 | 22.48 | 89.19 | 22.8 | 98.49% |
|  | PYCN09 | 24.33 | 90.17 | 24.68 | 98.51% |
|  | PYCN10 | 22.19 | 89.2 | 22.54 | 98.62% |
| *Coilia nasus taihuensis* | BMHCNT01 | 28.3 | 91.03 | 28.16 | 98.55% |
|  | BMHCNT02 | 31.6 | 90.15 | 31.13 | 98.62% |
|  | BMHCNT03 | 24.09 | 89.04 | 23.94 | 98.54% |
|  | BMHCNT04 | 17.31 | 87.75 | 17.06 | 98.90% |
|  | BMHCNT05 | 25.47 | 88.88 | 25.22 | 98.49% |
|  | BMHCNT06 | 22.89 | 88.74 | 22.76 | 98.50% |
|  | BMHCNT07 | 32.56 | 90.11 | 32.48 | 98.65% |
|  | BMHCNT08 | 22.28 | 89.18 | 22.11 | 98.41% |
|  | BMHCNT09 | 21.81 | 88.3 | 21.52 | 98.54% |
|  | BMHCNT10 | 30.4 | 89.67 | 29.99 | 98.54% |
|  | CHCNT01 | 20.96 | 88.82 | 20.87 | 98.48% |
|  | CHCNT02 | 28.04 | 90.4 | 28.08 | 98.51% |
|  | CHCNT03 | 22.34 | 88.78 | 22.49 | 98.51% |
|  | CHCNT04 | 29.78 | 91.66 | 29.91 | 98.44% |
|  | CHCNT05 | 24.37 | 89.45 | 24.57 | 98.48% |
|  | CHCNT06 | 25.55 | 89.47 | 25.61 | 98.37% |
|  | CHCNT08 | 23.78 | 89.1 | 23.82 | 98.46% |
|  | CHCNT09 | 28.54 | 89.52 | 28.58 | 98.49% |
|  | CHCNT10 | 19.83 | 88.54 | 19.88 | 98.39% |
|  | DPHCNT01 | 24.02 | 88.69 | 23.86 | 98.40% |
|  | DPHCNT02 | 27.59 | 90.83 | 27.25 | 98.35% |
|  | DPHCNT03 | 23.04 | 88.44 | 22.86 | 98.39% |
|  | DPHCNT04 | 24.11 | 89.1 | 23.83 | 98.36% |
|  | DPHCNT05 | 25.16 | 88.91 | 24.98 | 98.44% |
|  | DPHCNT06 | 24.94 | 89.23 | 24.77 | 98.37% |
|  | DPHCNT07 | 25.08 | 90.71 | 24.91 | 98.46% |
|  | DPHCNT08 | 30.75 | 91.33 | 30.69 | 98.52% |
|  | DPHCNT09 | 24.74 | 89.04 | 24.62 | 98.36% |
|  | DPHCNT10 | 29.65 | 91.48 | 29.34 | 98.43% |
|  | GHCNT01 | 20.55 | 88.3 | 20.68 | 98.37% |
|  | GHCNT02 | 21.67 | 88.22 | 21.67 | 98.30% |
|  | GHCNT03 | 20.35 | 88.42 | 20.44 | 98.32% |
|  | GHCNT04 | 20.55 | 88.27 | 20.69 | 98.44% |
|  | GHCNT05 | 25.6 | 89.37 | 25.64 | 98.34% |
|  | GHCNT06 | 28.68 | 89.45 | 28.71 | 98.42% |
|  | GHCNT07 | 24.96 | 89.95 | 24.99 | 98.34% |
|  | GHCNT08 | 24.83 | 91.84 | 24.79 | 98.41% |
|  | GHCNT09 | 20.68 | 88.64 | 20.53 | 98.64% |
|  | GHCNT11 | 22.87 | 90.27 | 22.97 | 98.33% |
|  | GHCNT12 | 23.83 | 90.63 | 24.08 | 98.36% |
|  | GHCNT13 | 22.02 | 90.38 | 22.22 | 98.34% |
|  | GHCNT14 | 23.52 | 90.68 | 23.7 | 98.33% |
|  | GHCNT15 | 23.28 | 90.86 | 23.5 | 98.41% |
|  | GHCNT16 | 21.12 | 90.5 | 21.18 | 98.45% |
|  | GHCNT17 | 22.79 | 90.55 | 22.97 | 98.11% |
|  | GHCNT18 | 21.61 | 90.31 | 21.46 | 98.25% |
|  | GHCNT19 | 22.96 | 90.77 | 23.07 | 98.39% |
|  | GHCNT20 | 23.95 | 90.53 | 23.87 | 98.23% |
|  | GYHCNT01 | 22.22 | 91.34 | 22.18 | 98.27% |
|  | GYHCNT02 | 22.98 | 91.68 | 22.79 | 98.36% |
|  | GYHCNT03 | 21.34 | 91.66 | 21.35 | 98.43% |
|  | GYHCNT04 | 24.87 | 91.83 | 24.83 | 98.38% |
|  | GYHCNT05 | 24.34 | 91.33 | 24.27 | 98.13% |
|  | GYHCNT06 | 23.87 | 91.33 | 23.83 | 98.24% |
|  | GYHCNT07 | 26.9 | 92.23 | 26.66 | 98.42% |
|  | GYHCNT08 | 22.75 | 92.27 | 22.54 | 98.42% |
|  | GYHCNT09 | 23.17 | 91.48 | 23.21 | 98.28% |
|  | GYHCNT10 | 23.97 | 91.73 | 24.01 | 98.30% |
|  | HZHCNT01 | 23.11 | 88.9 | 22.97 | 98.22% |
|  | HZHCNT02 | 20.22 | 88.26 | 20.11 | 98.26% |
|  | HZHCNT03 | 22.62 | 88.91 | 22.43 | 98.37% |
|  | HZHCNT04 | 25.76 | 89.2 | 25.62 | 98.43% |
|  | HZHCNT05 | 29.67 | 91.64 | 29.5 | 98.40% |
|  | HZHCNT06 | 31.59 | 90.81 | 31.08 | 98.36% |
|  | HZHCNT07 | 24.4 | 89.06 | 24.31 | 98.44% |
|  | HZHCNT08 | 23.33 | 88.87 | 23.24 | 98.25% |
|  | HZHCNT09 | 26.47 | 88.65 | 26.14 | 98.24% |
|  | HZHCNT10 | 20.97 | 88.01 | 20.85 | 98.36% |
|  | LMHCNT01 | 23.53 | 91.34 | 22.38 | 95.15% |
|  | LMHCNT02 | 23.84 | 90.42 | 23.71 | 98.38% |
|  | LMHCNT03 | 25.61 | 90.6 | 25.5 | 98.33% |
|  | LMHCNT04 | 22.67 | 90.68 | 22.67 | 98.31% |
|  | LMHCNT05 | 20.85 | 89.86 | 20.76 | 98.35% |
|  | LMHCNT06 | 21.93 | 91.02 | 21.98 | 98.17% |
|  | LMHCNT07 | 23.49 | 91.48 | 23.31 | 98.36% |
|  | LMHCNT08 | 24.76 | 92.08 | 24.59 | 98.34% |
|  | LMHCNT09 | 23.37 | 91.99 | 23.19 | 98.40% |
|  | LMHCNT10 | 23.62 | 91.45 | 23.31 | 98.34% |
|  | THCNT01 | 22.72 | 89.88 | 22.74 | 98.07% |
|  | THCNT04 | 25.32 | 89.58 | 25.49 | 98.17% |
|  | THCNT05 | 21.53 | 90.69 | 21.56 | 97.95% |
|  | THCNT10 | 21.99 | 89.21 | 22.11 | 98.06% |
|  | THCNT11 | 21.87 | 90.46 | 21.98 | 98.09% |
|  | THCNT12 | 23.91 | 89.36 | 23.97 | 98.25% |
|  | THCNT13 | 22.19 | 90.87 | 22.38 | 98.19% |
|  | THCNT14 | 22.05 | 91.34 | 22.16 | 98.17% |
|  | THCNT17 | 21.47 | 88.94 | 21.63 | 97.99% |
|  | THCNT20 | 21.47 | 90.76 | 21.56 | 98.09% |

**Table S2. The genetic differentiation index (*F*st) among the 12 geographical populations of *Coilia nasus* covering the Yangtze River, Huaihe River and Yellow River system.**

| Population | AQ | PY | TZ | CM | TH | GH | CH | HZH | BMH | DPH | LMH |
| --- | --- | --- | --- | --- | --- | --- | --- | --- | --- | --- | --- |
| PY | 0.0010 | - |  |  |  |  |  |  |  |  |  |
| TZ | 0.0101 | 0.0118 | - |  |  |  |  |  |  |  |  |
| CM | 0.0350 | 0.0371 | 0.0087 | - |  |  |  |  |  |  |  |
| TH | 0.0371 | 0.0391 | 0.0227 | 0.0198 | - |  |  |  |  |  |  |
| GH | 0.0350 | 0.0367 | 0.0210 | 0.0177 | 0.00001 | - |  |  |  |  |  |
| CH | 0.0355 | 0.0381 | 0.0255 | 0.0267 | 0.0212 | 0.0127 | - |  |  |  |  |
| HZH | 0.0840 | 0.0864 | 0.0711 | 0.0696 | 0.0733 | 0.0628 | 0.0393 | - |  |  |  |
| BMH | 0.0992 | 0.1011 | 0.0859 | 0.0845 | 0.0889 | 0.0781 | 0.0528 | 0.0005 | - |  |  |
| DPH | 0.0946 | 0.0970 | 0.0813 | 0.0803 | 0.0850 | 0.0736 | 0.0508 | 0.0033 | 0.0028 | - |  |
| LMH | 0.1101 | 0.1122 | 0.0983 | 0.0996 | 0.1036 | 0.0955 | 0.0705 | 0.0104 | 0.0034 | 0.0088 | - |
| GYH | 0.0935 | 0.0953 | 0.0803 | 0.0787 | 0.0819 | 0.0704 | 0.0490 | 0.0030 | 0.0013 | 0.0044 | 0.0062 |

**Table S3. Significantly enriched Gene Ontology (GO) terms of** **candidate genes exhibiting selection signals in anadromous *Coilia nasus.***

| GO term | Description | Gene number | *P* value |
| --- | --- | --- | --- |
| GO:0004947 | bradykinin receptor activity | 3 | 0.0000 |
| GO:0051499 | D-aminoacyl-tRNA deacylase activity | 3 | 0.0000 |
| GO:0002161 | aminoacyl-tRNA editing activity | 3 | 0.0000 |
| GO:0006939 | smooth muscle contraction | 3 | 0.0003 |
| GO:0008134 | transcription factor binding | 3 | 0.0007 |
| GO:0003700 | DNA-binding transcription factor activity | 17 | 0.0030 |
| GO:0006950 | response to stress | 3 | 0.0040 |
| GO:0005328 | neurotransmitter: sodium symporter activity | 4 | 0.0040 |
| GO:0006355 | regulation of transcription, DNA-templated | 26 | 0.0044 |
| GO:0043565 | sequence-specific DNA binding | 15 | 0.0045 |
| GO:0003677 | DNA binding | 24 | 0.0057 |
| GO:0000724 | double-strand break repair via homologous recombination | 2 | 0.0070 |
| GO:0006836 | neurotransmitter transport | 4 | 0.0078 |
| GO:0046872 | metal ion binding | 10 | 0.0081 |
| GO:0015914 | phospholipid transport | 3 | 0.0095 |
| GO:0019773 | proteasome core complex, alpha-subunit complex | 2 | 0.0133 |
| GO:0051056 | regulation of small GTPase mediated signal transduction | 2 | 0.0158 |
| GO:0000183 | chromatin silencing at rDNA | 1 | 0.0164 |
| GO:0001817 | regulation of cytokine production | 1 | 0.0164 |
| GO:0003382 | epithelial cell morphogenesis | 1 | 0.0164 |
| GO:0003721 | telomerase RNA reverse transcriptase activity | 1 | 0.0164 |
| GO:0003964 | RNA-directed DNA polymerase activity | 1 | 0.0164 |
| GO:0004645 | phosphorylase activity | 1 | 0.0164 |
| GO:0005720 | nuclear heterochromatin | 1 | 0.0164 |
| GO:0006629 | lipid metabolic process | 2 | 0.0164 |
| GO:0006213 | pyrimidine nucleoside metabolic process | 1 | 0.0164 |
| GO:0010825 | positive regulation of centrosome duplication | 1 | 0.0164 |
| GO:0022011 | myelination in peripheral nervous system | 1 | 0.0164 |
| GO:0030194 | positive regulation of blood coagulation | 1 | 0.0164 |
| GO:0030277 | maintenance of gastrointestinal epithelium | 1 | 0.0164 |
| GO:0034316 | negative regulation of Arp2/3 complex-mediated actin nucleation | 1 | 0.0164 |
| GO:0040001 | establishment of mitotic spindle localization | 1 | 0.0164 |
| GO:0047710 | bis(5-adenosyl)-triphosphatase activity | 1 | 0.0164 |
| GO:0050688 | regulation of defense response to virus | 1 | 0.0164 |
| GO:0051764 | actin crosslink formation | 1 | 0.0164 |
| GO:0071933 | Arp2/3 complex binding | 1 | 0.0164 |
| GO:0006811 | ion transport | 2 | 0.0185 |
| GO:0004012 | phospholipid-translocating ATPase activity | 2 | 0.0214 |
| GO:0017048 | Rho GTPase binding | 2 | 0.0244 |
| GO:0003884 | D-amino-acid oxidase activity | 1 | 0.0325 |
| GO:0004040 | amidase activity | 1 | 0.0325 |
| GO:0004462 | lactoylglutathione lyase activity | 1 | 0.0325 |
| GO:0004822 | isoleucine-tRNA ligase activity | 1 | 0.0325 |
| GO:0004994 | somatostatin receptor activity | 1 | 0.0325 |
| GO:0006306 | DNA methylation | 1 | 0.0325 |
| GO:0006428 | isoleucyl-tRNA aminoacylation | 1 | 0.0325 |
| GO:0007270 | neuron-neuron synaptic transmission | 1 | 0.0325 |
| GO:0009008 | DNA-methyltransferase activity | 1 | 0.0325 |
| GO:0009887 | animal organ morphogenesis | 1 | 0.0325 |
| GO:0016717 | oxidoreductase activity | 1 | 0.0325 |
| GO:0032088 | negative regulation of NF-kappaB transcription factor activity | 1 | 0.0325 |
| GO:0033209 | tumor necrosis factor-mediated signaling pathway | 1 | 0.0325 |
| GO:0046416 | D-amino acid metabolic process | 1 | 0.0325 |
| GO:0048251 | elastic fiber assembly | 1 | 0.0325 |
| GO:0090307 | mitotic spindle assembly | 1 | 0.0325 |
| GO:0004576 | oligosaccharyl transferase activity | 1 | 0.0483 |
| GO:0005763 | mitochondrial small ribosomal subunit | 1 | 0.0483 |
| GO:0006596 | polyamine biosynthetic process | 1 | 0.0483 |
| GO:0005509 | calcium ion binding | 1 | 0.0483 |
| GO:0016493 | C-C chemokine receptor activity | 1 | 0.0483 |
| GO:0016813 | hydrolase activity | 1 | 0.0483 |
| GO:0045214 | sarcomere organization | 1 | 0.0483 |
| GO:0045786 | negative regulation of cell cycle | 1 | 0.0483 |
| GO:0090162 | establishment of epithelial cell polarity | 1 | 0.0483 |
| GO:0019904 | protein domain specific binding | 2 | 0.0498 |

**Table S4. Significantly enriched KEGG pathways of** **candidate genes exhibiting selection signals in anadromous *Coilia nasus.***

| Pathway ID | Description | Gene number | *P* value |
| --- | --- | --- | --- |
| ko04212 | Longevity regulating pathway - worm | 8 | 0.0017 |
| ko00480 | Glutathione metabolism | 5 | 0.0085 |
| ko00470 | D-Amino acid metabolism | 2 | 0.0107 |
| ko00982 | Drug metabolism - cytochrome P450 | 4 | 0.0134 |
| ko03050 | Proteasome | 4 | 0.0303 |
| ko04978 | Mineral absorption | 4 | 0.0469 |

**Table S5. Significantly enriched Gene Ontology (GO) terms candidate genes exhibiting selection signals in freshwater-resident *Coilia nasus taihuensis.***

| GO term | Description | Gene number | *P* value |
| --- | --- | --- | --- |
| GO:0045211 | postsynaptic membrane | 8 | 0.000 |
| GO:0022848 | acetylcholine-gated cation-selective channel activity | 7 | 0.000 |
| GO:0005886 | plasma membrane | 18 | 0.000 |
| GO:0015057 | thrombin-activated receptor activity | 4 | 0.000 |
| GO:0070493 | thrombin-activated receptor signaling pathway | 4 | 0.000 |
| GO:0022857 | transmembrane transporter activity | 13 | 0.000 |
| GO:0005230 | extracellular ligand-gated ion channel activity | 10 | 0.000 |
| GO:0034220 | ion transmembrane transport | 10 | 0.000 |
| GO:0007596 | blood coagulation | 5 | 0.001 |
| GO:0043124 | negative regulation of I-kappaB kinase/NF-kappaB signaling | 2 | 0.001 |
| GO:0047390 | glycerophosphocholine activity | 2 | 0.001 |
| GO:0007156 | homophilic cell adhesion via plasma membrane adhesion molecules | 13 | 0.001 |
| GO:0008083 | growth factor activity | 10 | 0.003 |
| GO:0090286 | cytoskeletal anchoring at nuclear membrane | 3 | 0.003 |
| GO:0045786 | negative regulation of cell cycle | 2 | 0.004 |
| GO:0005104 | fibroblast growth factor receptor binding | 4 | 0.004 |
| GO:0008543 | fibroblast growth factor receptor signaling pathway | 4 | 0.006 |
| GO:0030001 | metal ion transport | 4 | 0.007 |
| GO:0050727 | regulation of inflammatory response | 2 | 0.007 |
| GO:0051082 | unfolded protein binding | 5 | 0.011 |
| GO:0004800 | thyroxine 5-deiodinase activity | 2 | 0.012 |
| GO:0015075 | ion transmembrane transporter activity | 2 | 0.012 |
| GO:0046982 | protein heterodimerization activity | 2 | 0.012 |
| GO:0070373 | negative regulation of ERK1 and ERK2 cascade | 2 | 0.012 |
| GO:0006811 | ion transport | 18 | 0.012 |
| GO:0008237 | metallopeptidase activity | 7 | 0.013 |
| GO:0005215 | transporter activity | 8 | 0.013 |
| GO:0005216 | ion channel activity | 16 | 0.016 |
| GO:0007219 | Notch signaling pathway | 4 | 0.017 |
| GO:0007155 | cell adhesion | 16 | 0.019 |
| GO:0002755 | MyD88-dependent toll-like receptor signaling pathway | 2 | 0.031 |
| GO:0003725 | double-stranded RNA binding | 2 | 0.031 |
| GO:0005635 | nuclear envelope | 2 | 0.031 |
| GO:0000256 | allantoin catabolic process | 1 | 0.036 |
| GO:0000836 | Hrd1p ubiquitin ligase complex | 1 | 0.036 |
| GO:0001787 | natural killer cell proliferation | 1 | 0.036 |
| GO:0001866 | NK T cell proliferation | 1 | 0.036 |
| GO:0003847 | 1-alkyl-2-acetylglycerophosphocholine esterase activity | 1 | 0.036 |
| GO:0004037 | allantoicase activity | 1 | 0.036 |
| GO:0004362 | glutathione-disulfide reductase activity | 1 | 0.036 |
| GO:0004726 | non-membrane spanning protein tyrosine phosphatase activity | 1 | 0.036 |
| GO:0006689 | ganglioside catabolic process | 1 | 0.036 |
| GO:0006749 | glutathione metabolic process | 1 | 0.036 |
| GO:0007292 | female gamete generation | 1 | 0.036 |
| GO:0008156 | negative regulation of DNA replication | 1 | 0.036 |
| GO:0008420 | CTD phosphatase activity | 1 | 0.036 |
| GO:0009612 | response to mechanical stimulus | 1 | 0.036 |
| GO:0016428 | tRNA (cytosine-5-)-methyltransferase activity | 1 | 0.036 |
| GO:0017038 | protein import | 1 | 0.036 |
| GO:0019408 | dolichol biosynthetic process | 1 | 0.036 |
| GO:0030321 | transepithelial chloride transport | 1 | 0.036 |
| GO:0031704 | apelin receptor binding | 1 | 0.036 |
| GO:0032299 | ribonuclease H2 complex | 1 | 0.036 |
| GO:0034457 | Mpp10 complex | 1 | 0.036 |
| GO:0043024 | ribosomal small subunit binding | 1 | 0.036 |
| GO:0050868 | negative regulation of T cell activation | 1 | 0.036 |
| GO:0051169 | nuclear transport | 1 | 0.036 |
| GO:0060048 | cardiac muscle contraction | 1 | 0.036 |
| GO:0060315 | negative regulation of ryanodine-sensitive calcium-release channel activity | 1 | 0.036 |
| GO:0070940 | dephosphorylation of RNA polymerase II C-terminal domain | 1 | 0.036 |
| GO:0071013 | catalytic step 2 spliceosome | 1 | 0.036 |
| GO:1902475 | L-alpha-amino acid transmembrane transport | 1 | 0.036 |
| GO:1903779 | regulation of cardiac conduction | 1 | 0.036 |
| GO:1904423 | dehydrodolichyl diphosphate synthase complex | 1 | 0.036 |
| GO:0055085 | transmembrane transport | 29 | 0.036 |
| GO:0046856 | phosphatidylinositol dephosphorylation | 3 | 0.037 |
| GO:0006457 | protein folding | 5 | 0.037 |
| GO:0008047 | enzyme activator activity | 2 | 0.039 |
| GO:0030512 | negative regulation of transforming growth factor beta receptor signaling pathway | 2 | 0.039 |
| GO:0034993 | meiotic nuclear membrane microtubule tethering complex | 2 | 0.039 |
| GO:0046658 | anchored component of plasma membrane | 2 | 0.039 |
| GO:0004888 | transmembrane signaling receptor activity | 10 | 0.039 |
| GO:0016592 | mediator complex | 3 | 0.047 |
| GO:0004553 | hydrolase activity, hydrolyzing O-glycosyl compounds | 4 | 0.049 |
